# The Uses, Nutritional Advantages, and Challenges of Traditional Fermented Alcoholic Beverages for Indigenous Communities in Ethiopia

**DOI:** 10.1155/ijm/5679327

**Published:** 2026-05-06

**Authors:** Mulugeta Fentahun

**Affiliations:** ^1^ Department of Biology, College of Natural and Computational Sciences, Debre Markos University, Debre Markos, Amhara, Ethiopia, dmu.edu.et

**Keywords:** challenges, Ethiopia, fermenting microorganisms, traditional fermented beverages

## Abstract

Traditional fermented alcoholic beverages (TFABs) are an integral part of Ethiopian culture, serving as food and recreational sources and vital elements of social, religious, and ceremonial events. Ethiopia is known for its wide variety of TFABs, which are made using indigenous knowledge handed down through generations. *Tella*, *Borde*, *Shamita*, *Korefe*, *Cheka*, *Tej*, *Ogol*, and *Keribo* are among the most popular alcoholic beverages in Ethiopia. These beverages are often made with cereals and natural ingredients such as *gesho*, vegetables, spices, and herbs. TFABs provide essential nutrients, such as proteins, carbohydrates, vitamins, and minerals, and offer other functional health benefits, such as probiotic effects, improved digestibility, and increased nutrient bioavailability. Furthermore, the therapeutic potential of Ethiopian TFABs is enhanced by the presence of bioactive compounds and antioxidants, which may lower the risk of chronic illnesses. Despite their cultural and nutritional importance, TFAB production faces several challenges, such as informal brewing practices, quality control, commercialization, and safety assurance, a lack of standardization, possible contamination (e.g., acrylamide, methanol, and microbial contamination), a lack of regulatory oversight, and limited scientific attention. Ethiopian TFABs have significant growth potential if they receive adequate support in areas such as financial assistance, technical training, public policy, marketing assistance, business skills development, and the use of modern fermentation technology. This review summarizes Ethiopian TFABs′ cultural significance, nutritional advantages, and main obstacles of Ethiopian TFABs, emphasizing support services to promote traditional fermented beverages, preserve indigenous knowledge, and enhance public health.

## 1. Introduction

Traditional fermented alcoholic beverages (TFABs) have played a significant role in Ethiopian culture for centuries. They play a vital role in social, religious, and ceremonial events, in addition to being sources of food and entertainment [1, 2]. Ethiopia has one of the richest fermented traditional foods and alcoholic beverages in Africa [3]. Most countries have unique TFABs that are indigenous to their respective locations [4, 5]. *Tella*, *Tej*, *Areke*, *Keribo*, *Booka*, *Ogol*, *Bubegne*, *Korefe*, *Buqri*, *Shameta*, *Borde*, *Cheka*, *Imbushbush*, *Winetej*, *Duka*, *Suwa*, *Girawa*, *Jikita*, and *Birz* are among the known traditional alcoholic beverages in Ethiopia [6–14]. The production of TFABs employs age‐old fermentation techniques, often passed down from parent to child, and reflects both cultural traditions and indigenous knowledge systems [15]. Ethiopian TFABs made from cereals are particularly popular because of their nutritional content, flavor, and health benefits [16].

TFABs have the potential to significantly improve the quality of life of both rural and urban residents [17]. In developing countries like Ethiopia, TFABs serve many purposes. They are used for medical reasons, religious and nonreligious celebrations, weddings, recreational activities [2, 16], festivals, social events, funerals, treating strangers with respect, social gatherings, and arguments and as food alternatives [18]. The majority of Ethiopian TFABs are a good source of vital nutrients, such as carbohydrates, proteins, vitamins (particularly B complex vitamins), and minerals, such as calcium, iron, and zinc [19–21]. These traditional beverages offer an additional source of nutrition and energy in regions where access to balanced diets is limited, especially during periods of food scarcity, agricultural labor, and fasting [19]. TFABs also have many health advantages, such as increasing digestibility, enhancing the bioavailability and absorbability of certain nutrients, reducing antinutritional factors, and providing probiotic benefits to consumers [22, 23].

Ethiopian TFABs, especially those made with grains, *gesho* (*Rhamnus prinoides* L.), and natural herbs, are rich in bioactive compounds. These bioactive substances possess antioxidant properties that can help protect cells from oxidative stress and improve overall health [23–25]. According to recent research, Ethiopian TFABs have significant functional and therapeutic potential because of their potent antioxidants, rich bioactive substances, and beneficial microbial metabolites. These qualities may enhance general well‐being and lower the risk of developing chronic illnesses [26–28].

However, traditional brewing techniques are being challenged by urbanization and industrialization. Their development and recognition have been hindered by informal production, differences in fermentation processes, a lack of standardization, potential health risks (e.g., acrylamide, methanol, and microbial contamination), and limited scientific attention. Generally, household producers are not subject to government regulation, which restricts their access to quality control, training, appropriate storage conditions, and market opportunities [5, 29–31]. Ethiopian TFABs do not pay attention to the process and quality development because of a lack of backslopping, starting culture, and standardized procedures. However, they have not been commercialized yet, so the process has not been standardized and modernized [30]. Ethiopian traditional fermentation methods are primarily limited by their inefficiency, low yields, and inconsistent product quality. It has also been reported that the main issues facing TFABs include a lack of standardized procedure, proper logistics for scaling up to large production, knowledge of appropriate packaging materials and transport systems, institutional support mechanisms, branding and trademarks in manufactured products, market networking, and training skills [5, 28, 29]. In some cases, there are safety concerns related to bacterial pathogens associated with raw materials or unhygienic practices during processing [32]. Additionally, public health issues may limit the sale and distribution of TFABs, resulting in occasional conflicts between modern legal systems and traditional preservation practices [31].

Various TFABs have the potential to transform home‐based arts into modern industry necessities through research and technological modification and/or development [33]. Understanding the cultural applications, nutritional value, and primary challenges of TFABs in Ethiopia is essential for advancing local economies, preserving indigenous knowledge systems, and promoting public health [3, 28]. Although TFABs have long been consumed in Ethiopia and hold cultural significance, there is limited scientific research on their uses, nutritional value, and related challenges for indigenous communities. Thus, this review provides current scientific information on the cultural use, nutritional benefits, and associated challenges of TFABs for indigenous people in Ethiopia.

## 2. Uses, Types, and Nutritional Advantages of Traditional Fermented Beverages

### 2.1. Uses of Traditional Fermented Beverages

In Ethiopia, TFABs are used not only as refreshments but also as symbols of identity, culture, and social cohesion, demonstrating the strong bonds that exist between people, traditions, and group life [30, 34, 35]. They continue to contribute significantly to Ethiopia′s cultural heritage through collective celebrations, artisanal knowledge, and shared customs, bridging the past and present to preserve Ethiopian traditions. They play a major role in poverty reduction, sustainable development, and food security. TFABs are used for medical purposes, religious and nonreligious celebrations, leisure activities, and social events [2, 18, 35]. Their consumption is involved during religious gatherings, festivals, weddings, baptisms, funerals, mourning events, and community service like *debo*, *iqub* (one of the money‐saving methods), *edir* (a traditional mutual‐aid organization for the community), and *maheber*. These beverages, such as *meskel* and *timket*, are particularly important because they are shared communally during traditional ceremonies and holidays [32, 35, 36]. Their production has deep roots in the traditions of the people, where they play a significant sociocultural and economic role [36, 37]. The production and consumption of traditional alcohol in Ethiopia remain widespread, particularly among the rural and urban segments of society [15].

Traditional fermented drinks provide substantial nutritional benefits and generate income in both urban and rural areas, especially for women who manufacture and sell them as part of home‐based enterprises [30, 38]. In addition to their social and cultural value, TFABs support both urban and rural economies and aid in the preservation of ancestral knowledge, guaranteeing the continuity of Ethiopia′s brewing customs. Many indigenous beverages are made using traditional fermentation knowledge passed down through generations. Since women are usually the primary brewers, the making and sharing of these traditional beverages serve to strengthen social bonds, hospitality, and the transmission of gendered knowledge [15]. In both urban and rural communities, preparation methods are often taught within families, preserving traditional recipes and promoting cultural continuity [38].

Drinking together strengthens community bonds, promotes cultural traditions, and builds a sense of belonging among its members. In many Ethiopian homes, these drinks are used in ceremonies to bless the occasion, demonstrate respect, and promote social harmony [1]. TFABs are sometimes consumed in specific social settings. This consumption is used together in a community of consumers governed by the rules of conviviality, sociability, and sociality. They are often consumed when they are near the production site. Drinking settings serve as important meeting places for people to exchange vital and mundane information about their lives. All social strata consume fermented beverages, especially men, and demarcate the separation of leisure from work. Cultural norms strongly influence drinking patterns, and the age at which one is allowed to drink is significant. Sanctions against women who drink excessively or specific types of alcohol have also been reported [2, 35, 39].

### 2.2. Types of Traditional Alcoholic Beverages in Ethiopia

**Figure 1:**
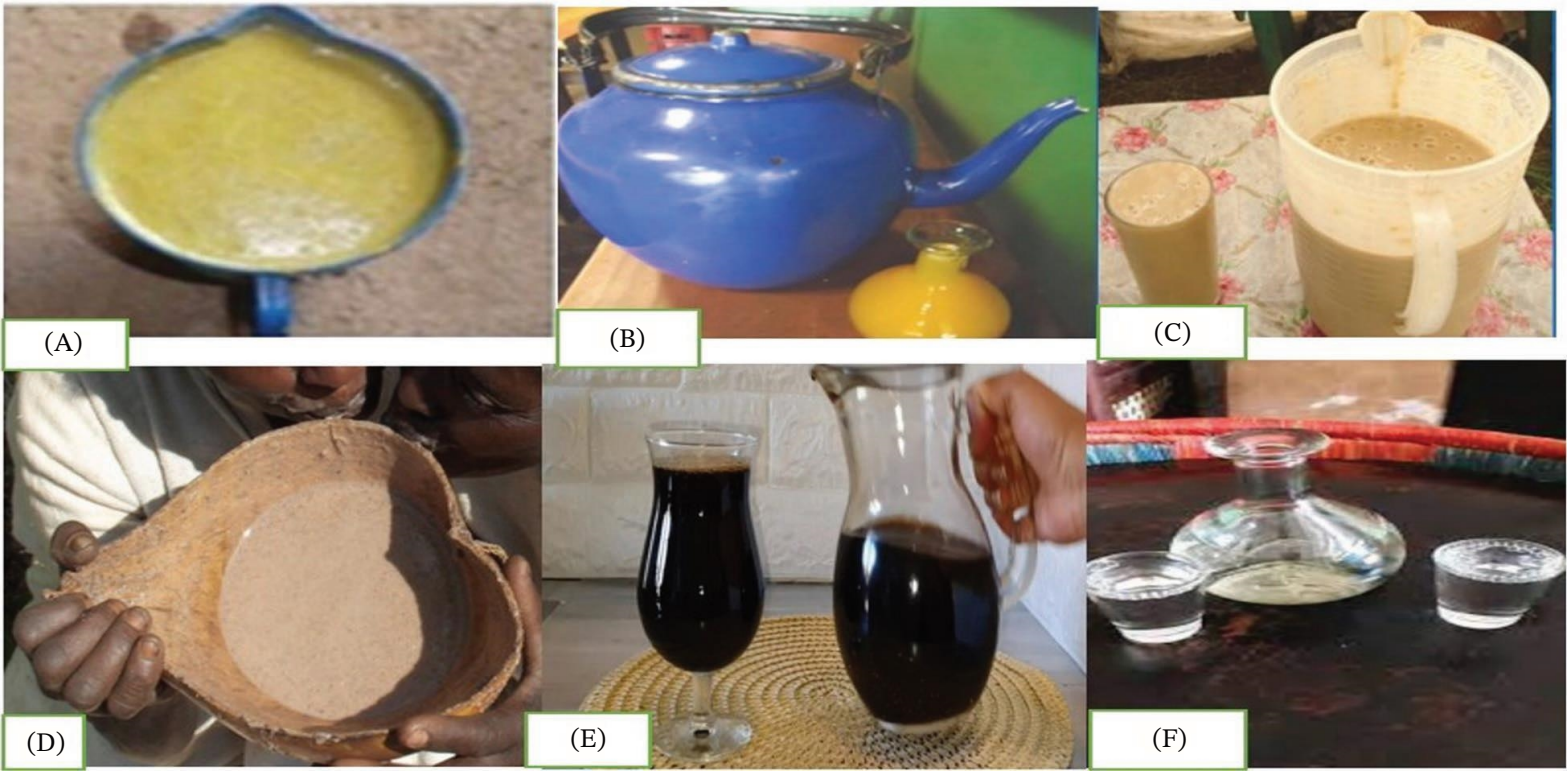
Different types of local Ethiopian alcoholic beverages: (A). Cheka, (B) Tej, (C) *Shamita*, (D) *Borde*, (E) *Keribo*, and (F) *Areke* [41–43].

Ethiopia has various traditional fermented drinks that are embedded in the country′s social life and culture. Local cereals, such as barley, maize, sorghum, finger millet, wheat, and teff, along with honey, vegetables, spices, and other natural ingredients, are frequently used to prepare these drinks. *Tella*, *Areke*, *Tej*, *Borde*, *Shamita*, *Korefe*, *Keribo*, *Ogol*, and *Cheka* are some of Ethiopia′s most popular traditional alcoholic beverages [7, 9–12, 40] (Figure 1; Table 1).

**Table 1:** Chemical composition of some of the traditionally fermented beverages in Ethiopia.

Product	Substrate	Nutritional composition	Microbes involved	pH	Consumption place	Reference
*Borde*	Maize (*Z. mays* L.), barley (*H. vulgare* L.), wheat (*T. aestivum* L.), finger millet (*E. coracana* L.), and sorghum (*S. bicolor* L.)	Total protein: 7.55%; fat: 6.88%; carbohydrates: 6.88%; ash: 3.66%; energy (Cal): 125.95	*Weissella confusa*, *Lactobacillus viridescens*, *Levilactobacillus spicheri*, *Levilactobacillus zymae*, *Limosilactobacillus pontis*, *Pediococcus pentosaceus*, *Saccharomyces* spp., *Pichia* spp., and *Rhodotorula* spp.	3.8	South Nation Nationality of Peoples (SNNP)	[12, 33, 44–46]
*Shamita*	Roasted barley (*H. vulgare* L.) flour, salt, linseed (*L. usitatissimum* L.) flour, and chili pepper (*C. annuum* L.)	Total protein: 10.37%; fat: 3.46%; ash: 6.58%; iron: 8.1 mg/100 g; zinc: 8.6 mg/100 g	*Lacticaseibacillus sharpeae*, *Lacticaseibacillus songhuajiangensis*, *Limosilactobacillus pontis*, *Paucilactobacillus vaccinostercus*, *Leuconostoc falkenbergense*, *Levilactobacillus zymae*, *Saccharomyces* spp., and *Rhodotorula* spp.	4.0	Addis Ababa and South Nation Nationality of Peoples (SNNP)	[35, 44, 47–49]
*Korefe*	Dehusked barley (*H. vulgare* L.) and “*gesho*” (*R. prinoides* L.)	Total protein: 7.3%; fat: 6.99%; carbohydrates: 2.31%; ash: 4.8%; energy (Cal): 101.35	*Saccharomyces* spp., *Lactobacillus* spp., *Lactococcus* spp., *Pediococcus* spp., *Enterococcus* spp., *Weissella* spp., Enterobacteriaceae, *Bacillus* spp., and *Micrococcus* spp.	4.0	Amhara and Tigray	[11, 46, 50]
*Booka*	Honey and the bark of the native tree “*Mange*” (*B. unijungata* L.)	Total protein: 8.8%; fat: 2.33%; carbohydrates: 5.47%; ash: 0.97%; energy (Cal): 78.05	—	3.01	South Ethiopia and the Guji community	[8, 43, 46]
*Bubugn*	Millet (*E. coracana* L.), sorghum (*S. bicolor* L.), barley (*H. vulgare* L.), and baker′s yeast (dry active yeast)	Fat: 6.67%; ash: 4.47%	Total mesophilic aerobic bacteria, yeasts, molds, and lactic acid bacteria	4.20	North Gondar	[51]
*Cheka*	Sorghum (*S. bicolor* L.), maize (*Z. mays*), finger millet (*E. coracana* L.), vegetables, root of taro (*C. esculenta* L.), and moringa (*M. oleifera*).	Total protein: 3.83%; fat: 1.49%; carbohydrates: 16.59%; ash: 0.79%; energy (Cal): 94.60; Ca: 13.95 mg/100 g; Fe: 20.76 mg/100 g; Zn: 0.95 mg/100 g; energy (Cal): 98.73	*Limosilactobacillus panis*, *Limosilactobacillus fermentum*, *Pediococcus pentosaceus*, *Lactobacillus delbrueckii*, *Saccharomyces* spp., *Candida* spp., and *Pichia* spp.	4.11	South nation nationality of peoples (SNNP)	[42, 44, 52, 53]
*Keribo*	Barley (*H. vulgare* L.), sugar, and bakery yeast (*S. cerevisiae*)	Fat: 2.5%; carbohydrates: 12.5%; Ca: 12.06 mg/100 g; Fe: 0.03 mg/100 g; Zn: 3.65 mg/100 g; Mg: 4.03 mg/100 g; energy (Cal): 94.60	*Leuconostoc mesenteroides*, *Leuconostoc falkenbergense*, *Liquorilactobacillus nagelii*, Aerobic mesophilic bacteria, and *Saccharomyces cerevisiae*	4.2	Amhara, Oromia, and Addis Ababa	[9, 44, 54]
*Tella*	Barley (*H. vulgare* L.), wheat (*T. aestivum* L.), maize (*Z. mays* L.), finger millet (*E. coracana* L.), sorghum (*S. bicolor* L.), “teff” (*E. tef* L.), and “*gesho*” (*R. prinoides* L.)	Total protein: 0.4%; fat: 0.0%; carbohydrates: 1.98%; calcium: 9.4 mg/100 g; iron: 8.1 mg/100 g; zinc: 8.6 mg/100 g; magnesium: 6.1 mg/100 g; potassium: 9.0 mg/100 g; sodium: 2.3 mg/100 g; niacin: 0.02/100 g; folate: 0.093/100 g	*Saccharomyces cerevisiae*, *Saccharomyces carlsbergensis*, *Lactobacillus pastorianum*, *Lactobacillus postanumi*, *Lactiplantibacillus* spp., *Paucilactobacillus* spp., *Peribacillus* spp., *Levilactobacillus* spp., *Lactobacillus* spp., *Companilactobacillus* spp., *Lacticaseibacillus* spp., *Acetobacter* spp., and *Paenibacillus* spp.	3.87–4.67	Amhara, Tigray, Oromia, Addis Ababa, and South Nation Nationality of Peoples (SNNP)	[5, 40, 44, 55, 56]

#### 2.2.1. *Tella*

**Figure 2:**
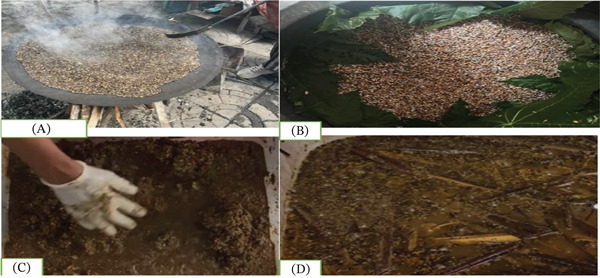
Raw materials for *Tella* and *Areke* preparation: (A) barley malt roasting on *biret mitad*, (B) sprouted barley on castor leaves, and (C, D) traditional *Tella* and *Areke* raw materials during fermenting [59].

It is one of the most popular traditional Ethiopian beers. *Tella* is made from a variety of grains, including barley, sorghum, teff, millet, wheat, maize, and *gesho* (*R. prinoides* L.) [5]. Similar to hops in Western beer, the unique leaf called *gesho* (*R. prinoides* L.) is used as a natural flavoring, with antibacterial properties, and a fermentation agent [57]. *Tella* is an opaque alcoholic beverage with a color range of light yellow to dark brown, an ethanol content of 3.98%–6.48% (*v*/*v*), and a pH of 3.87–4.67 [55]. Another kind of beverage is filter *Tella*, which is made similarly to regular *Tella* but is filtered to remove solid residues and is more concentrated [32]. Depending on the ethnic group, financial situation, and customs, different raw materials and production techniques are used to make *Tella*. There are several different names for *Tella* depending on the region, including Gurage′s *Tella*, Oromo′s *Tella*, and Amhara′s *Tella* (Figure 2) [19, 58]. Amhara *Tella* stands out due to its relatively high ethanol content and *gesho* (*R. prinoides* L.) composition. Gurage *Tella* is delicately aromatized with a variety of spices. *Gesho* is an antimicrobial and bittering ingredient commonly used in Amhara and Gurage recipes but not used in Oromo *Tella*. The preparation methods, ingredients, and flavor characteristics of Oromo *Tella* differ from those of Amhara or Gurage *Tella* [19].

**Figure 3:**
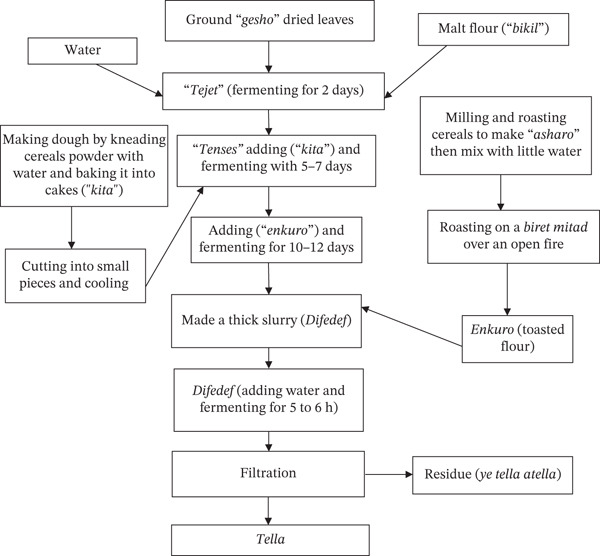
Flow diagram of the traditional *Tella* production method [60, 61].

**Figure 4:**
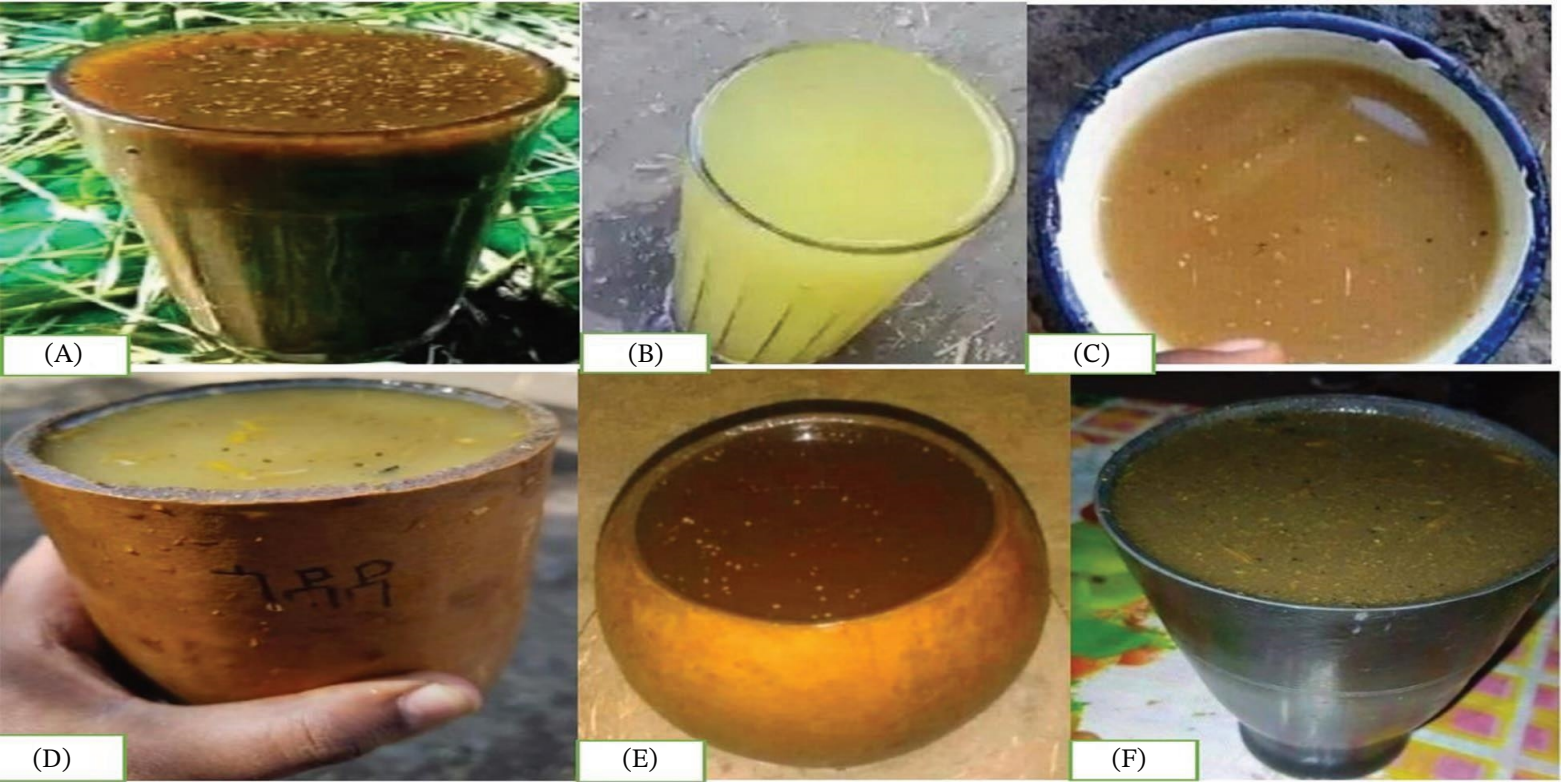
*Tella* varieties found in various Ethiopian regions: (A) Amhara′s *Tella* in a glass, (B) Oromo′s *Tella* in a glass, (C) Amhara′s *Tella* in a cup, (D) Gurage′s *Tella* in a gourd, (E) Amhara′s *Tella* in a gourd, and (F) Amhara′s *Tella* in a clay cup [58, 59].

Before the *Tella* fermentation process, fermentation jars (*gan*) are cleaned with *grawa* (*V. amygdalina* L.) and smoked with *weira* (*O. europaea L. cuspidate* L.) [60]. The main phases of the *Tella* fermentation process are *tejet*, *tenses*, and *difdif*, each distinguished by the addition of ingredients at various periods (Figure 3) [5]. To make a malt (*bikil*), the barley is soaked in water first for about 24 h at room temperature. The moistened grain is covered using fresh banana or castor leaves after 24 h, and it is then stored for three more days in a dry location (Figure 4) [10]. The germinated barley grain is then sun‐dried and ground into malt flour. The leaves and stems of *gesho* (*R. prinoides* L.) are simultaneously sun‐dried and crushed. Then, *bikil* flour and *gesho* powder are mixed with enough water in a clean traditional bioreactor called *insera*. *Tejet* is produced by allowing this mixture to ferment for 2 days [5]. A dough is made by mixing equal amounts of millet, sorghum, and teff flour with water. After that, the dough is cooked to make unleavened bread called locally as *ye Tella kita* [60]. This bread is then cut into pieces and mixed with the previously made *tejet.* After that, the mixture is firmly sealed and allowed anaerobic fermentation for 5–7 days so as to produce *tenses* [40]. Maize grain is soaked in water for about 3 days, after that dried, roasted, and ground to produce *enkuro*, a black maize flour that is the primary component that gives *Tella* its color [62]. The previously made *tenses* are then mixed with *enkuro* and anaerobically fermented for 10–12 days. Following this fermenting phase, a thick mixture known locally as *difdif* is made. After adding water, *difdif* is allowed to ferment for a further 5–6 h. Finally, solid leftovers are filtered out and given to consumers as *Tella* [62]. The shelf life of *Tella* appeared to be short, ranging from 5 to 7 days [5].

*Tella* is traditionally made at home using traditional recipes that have been handed down through generations, particularly by women [2]. Traditional *Tella* fermentation is a spontaneous and uncontrollable process that involves the natural growth of microorganisms. Raw materials, equipment, and utensils are sources of microorganisms used in the fermentation process [60, 63]. *Tella* is served at religious feasts, weddings, holidays, and community events and is an important part of Ethiopian life. It also goes along with cultural events like *idir* (community associations for mutual aid) and *mahber* (religious fellowship gatherings). *Tella* houses, also called *tellabets*, serve as neighborhood pubs in rural and semiurban areas where people gather to drink, interact with each other, and share news [3].

*Tella* is produced through spontaneous fermentation by complex and dynamic microbial consortia. Lactic acid bacteria (LABs), yeasts, and, in the early phases, different aerobic mesophilic bacteria (AMBs) and Enterobacteriaceae dominate the fermentation microbiota [40, 56, 60]. The *Saccharomyces*, *Lactobacillus*, and *Acetobacter* species are the predominant fermenting microorganisms in *Tella* [5]. The fermenting microorganisms in *Tella* are composed of *Saccharomyces* species (*Saccharomyces cerevisiae* and *S. carlsbergensis*) and *Lactobacillus* species, such as *L. pastorianum* and *L. postanumi*, which are frequently described [5, 56]. Yeasts dominate the fermenting flora after the end of the first stage until the completion of fermentation [60]. According to Sanz‐López et al. [44], the LAB community is characterized by a diverse species, including *Lactiplantibacillus*, *Paucilactobacillus*, *Peribacillus*, *Levilactobacillus*, *Lactobacillus*, *Companilactobacillus*, and *Lacticaseibacillus*. In addition to LABs, other bacterial species, such as *Acetobacter* and *Paenibacillus*, have also been detected in *Tella* samples.

#### 2.2.2. *Areke*

**Figure 5:**
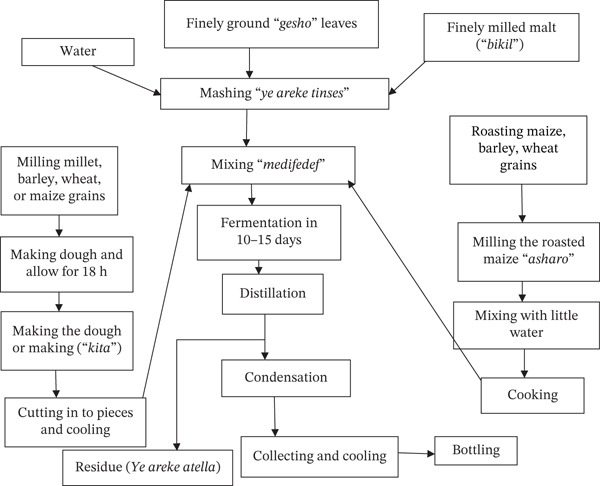
Flow sheet of the traditional *Areke* production method [65].

*Areke* is a traditional distilled alcoholic beverage made by distilling the fermented mash of various cereal grains (e.g., barley, wheat, maize, millet, sorghum, and teff) and *gesho* (*R. prinoides* L.) [10]. It is clear and colorless and has a higher alcohol content [64]. The range of alcohol content was 30% to nearly 50% (*v*/*v*) [55]. *Areke* is extremely potent, harmful to consume, and popular in small towns and rural areas. *Areke* can be compared to gin or vodka in terms of strength. Fermentation and distillation are the two main processing stages in the *Areke* production process. After fermentation, *Areke* is separated and concentrated using heat distillation. This gives the drink a high ethanol content compared to other fermented beverages. The traditional fermentation process of *Areke* relies on spontaneous, natural fermentation without intentional commercial yeast inoculation. The three fundamental steps in the *Areke* production process are *yereki-tensis*, *medifedef*, and *Areke*. *Yereki-tensis* was prepared by mixing *gesho* powder, *bikil* malt, and water in a fermenter and allowing it to ferment for a week (Figure 5). *Kita* (thin pancake‐like bread) and *enkuro* (toasted flour) were made from barley, millet, wheat, and maize. *Kita* was baked on a *mitad* after mixing water with flour from barley, wheat, and maize to form a dough, whereas *enkuro* was made by soaking, drying, and roasting maize flour on a *bret mitad* at 70°C–100°C. *Enkuro* and *kita* were added to *yereki-tensis* and fermented anaerobically for 15–20 days to produce *medifedef*. After fermentation was complete, the fermented mash was boiled for the *Areke* distillation process. The shelf life of *Areke* appears to be up to 1–2 years at room temperature [10, 64, 66].

**Figure 6:**
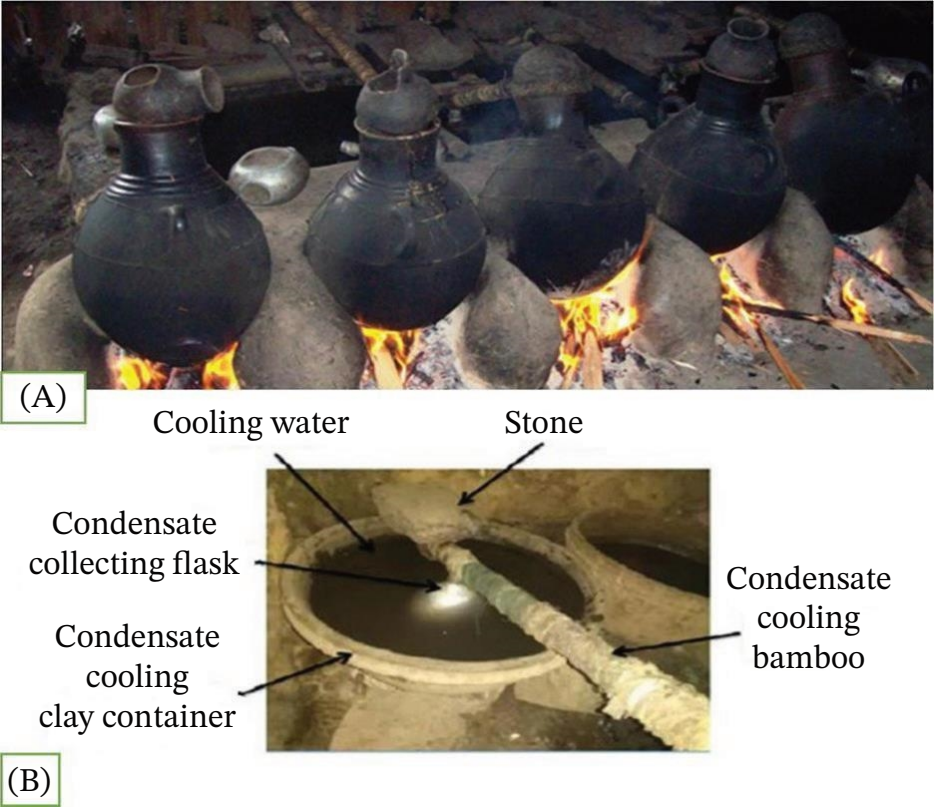
Local *Areke* distillation. (A) Traditional extraction of local *Areke*. (B) Process of traditional condensation [65].

*Areke* is traditionally divided into two categories: *terra-Areke* and *dagim-Areke*. “*Terra*” means “ordinary” in Amharic, while “*dagim*” means “second time,” indicating that it has undergone a second distillation. Redistilling the *Areke* increases its alcohol content. The alcohol content of *dagim-Areke* is approximately 45%. According to reports, *terra-Areke* has an alcohol content of 34.09% (*v*/*v*) [55, 66]. A clay pot, pot lid, condensation tube, and collecting flask were used during *Areke* distillation (Figure 6) [65]. The fermented mash was boiled to release vapors in a small clay pot called a *madiga*. The cloth was used to seal the *madiga* and prevent vapor loss. A hollow tube composed of dry bamboo (*Bambusa vulgaris* L.) carried the *Areke* vapor from a clay pot (boiler) to the collector. *Koda* was used as a collector, and vapor in *Koda* turned into liquid in the cooling water that was contained in the clay *tofaa* [67].

*Areke* is frequently drunk at special events like neighborhood get‐togethers, regional rituals, group festivities, and customary medical treatments. It is frequently served during festive occasions or shared as a gesture of hospitality among friends and family. Despite its strength, *Areke* is highly valued for its stimulating properties and cultural significance as a handcrafted spirit that symbolizes ingenuity and craftsmanship [1, 38]. *Areke* drinkers are mostly from lower socioeconomic classes, who are unable to purchase commercially produced alcoholic beverages [55, 68].

#### 2.2.3. *Tej*

**Figure 7:**
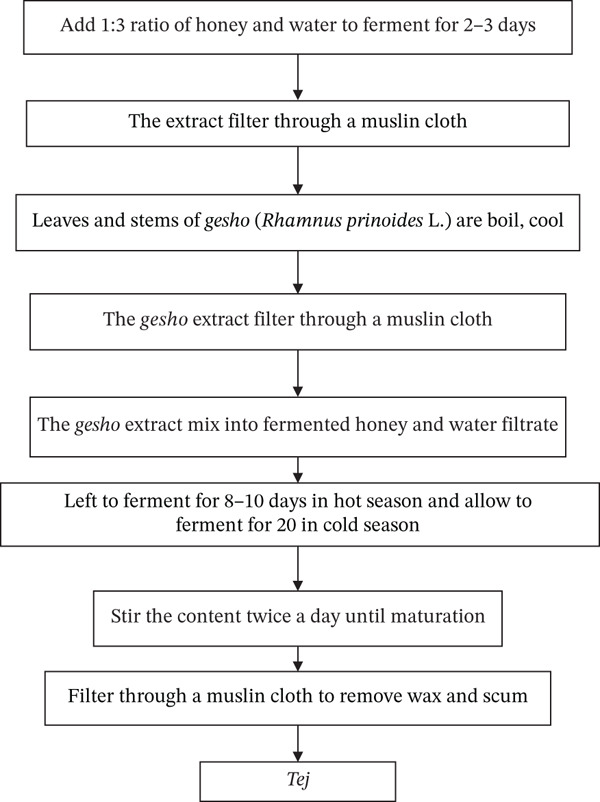
Flow chart of the *Tej* fermentation process [70].

*Tej* (honey wine) is one of the most popular and unique alcoholic beverages in Ethiopia. Depending on the fermentation time, *Tej* can have a sweet to semisweet flavor and a bright yellow or golden color. Typically, the alcohol content ranges between 7% and 11% (*v*/*v*) [69]. The *Tej* making process begins by cleaning the traditional fermenting container with water and fresh *grawa* (*V. amygdalina* L.) leaves and then smoking it using dried stems of *weyira* (*O. europaea* subsp. *cuspidate* L.). After allowing honey and water to ferment for 2–3 days in a 1:3 ratio, the extract was filtered through a muslin cloth (Figure 7). Leaves and stems of *gesho* (*R. prinoides* L.) are boiled, cooled, and mixed into the already fermented honey and water mixture. During the hot season, this mixture is left to ferment for 8–10 days, and during the cold season, it is allowed to ferment for 20 days [10, 70]. The product is ready to be served to customers in a distinctive glass known locally as *berele* after completing the fermentation period.

Traditional *Tej* fermentation relies on natural, spontaneous fermentation without commercial yeast inoculation. The process is started by native microbes that are present in raw materials, utensils, and the surrounding environment [71]. The type of honey used and the quantity of *gesho* (*R. prinoides* L.) added have a significant impact on the flavor of *Tej* [1]. *Tej* is estimated to have a shelf life of 1–3 months at room temperature and longer if stored in airtight containers; however, its quality may change over time [71]. *Tej*, which represents friendship and respect, is an important cultural component of Ethiopian hospitality. It is frequently consumed during weddings, religious festivals, social ceremonies, national holidays, and other happy occasions [72]. *Tej* is still made at home. In the past, it was served in royal courts and monasteries and was only offered to nobles, priests, and special visitors. *Tej bet*, or “houses of *Tej*,” are traditional taverns where people group together to drink, tell stories, and listen to traditional music [10].

*Tej* is produced through spontaneous fermentation by mixed and dynamic microbial consortia of LABs, yeasts, and other bacteria. The most common yeasts in *Tej* are *S. cerevisiae*, *Debaryomyces phaffii*, *Kluyveromyces bulgaricus*, and *Kluyveromyces veronae*, which are responsible for the overall *Tej f*ermentation dynamics [71]. The LABs in *Tej* are composed of *Leuconostoc*, *Pediococcus*, *Streptococcus*, and *Lactobacillus* species (mostly *Lactobacillus plantarum*). These bacteria are essential for producing acids, lowering pH, and improving the sensory quality of beverages [50, 71].

#### 2.2.4. *Borde*

*Borde* is a traditional fermented low‐alcohol beverage primarily consumed in southern and western Ethiopia. It is produced using simple equipment and spontaneous fermentation from various cereals, including teff, sorghum, finger millet, barley, wheat, and maize [12, 36]. *Borde* is a thick, opaque beverage with a sweet‐and‐sour flavor and a color ranging from whitish‐gray to brown [36, 73]. It has a low alcohol content (between 1% and 3%). It is frequently used as a meal substitute and beverage, particularly by low‐income households, farmers, and laborers who need energy. *Borde* has high protein, carbohydrate, vitamin, and mineral content, making it an essential source of nourishment. Local populations with low incomes may use up to 3 L of *Borde* every day [36].

**Figure 8:**
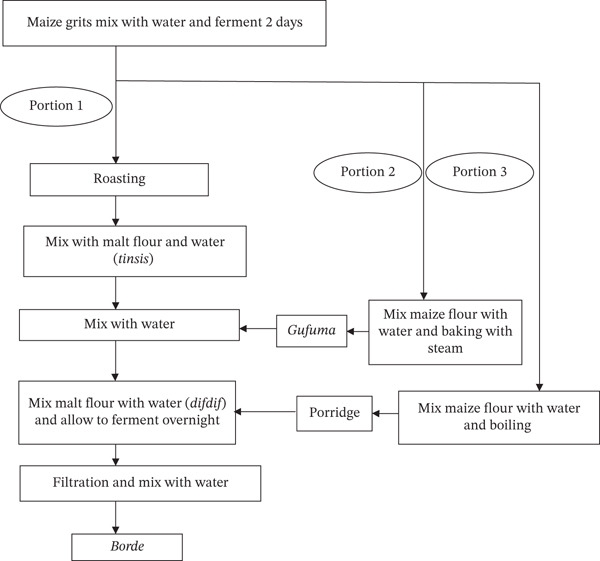
Flow chart of the *Borde* fermentation process [12, 36].

The process of making *Borde* starts when barley is soaked in water for about a day at room temperature to produce malt (*bikil*). The moistened grain is covered with new banana or castor leaves and stored for three more days in a dry location [10]. Malt flour is made after sun‐drying and grinding the germinated barley grain. A starter of approximately 1 L of *Borde* from a previous fermentation is sometimes added [12]. A proportionate amount of water was mixed with maize grits in parallel, and the mixture was fermented for 2 days (Figure 8). The fermented blend was divided into three portions. In portion one, “*enkuro*” is made by roasting approximately 40% of the mixture in a hot pan (*bret mitad*). The prepared “*enkuro*” is then mixed with malt flour and extra water, and it is left to ferment in the same mixing vessel (*gan*) for approximately 24 h [12, 36]. Fresh maize flour and water are mixed with the remaining 40% of the fermented maize grits. *Gafuma* is prepared by shaping this mixture into a ball and baking it with steam [36]. The thick brown mash locally known as *difdif* is then created by adding *gafuma* to previously made *tinsis*. The remaining 20% of the fermented maize grits were mixed with additional flour and water and heated to create a thick porridge. *Difdif* is then mixed with the prepared porridge, additional malt, and water. Finally, allow to ferment overnight. The mixture is filtered, and a small amount of water is added before serving to customers as *Borde* [73, 74]. The shelf life of *Borde* is no longer than 12 h at room temperature [36].

*Borde* enhances the breastfeeding experience. After giving birth, mothers are advised to consume a lot of it [54]. According to recent reports, the shelf life of *Borde* was extended when extracts of *Thymus schimperi* (*Tosign*) and *Moringa oleifera* (drumstick tree) were added [75]. *Borde* is also used for social and communal purposes; it is shared by family members, neighbors, and visitors during festivals, agricultural seasons, and informal gatherings [1].

The microbial fermentation of *Borde* is a complex and dynamic consortium of bacteria and yeasts that is crucial for alcohol production, flavor, and stability [36]. Among the bacterial population, LABs are predominant. These include *Weissella confusa*, *Lactobacillus viridescens*, *Levilactobacillus spicheri*, *Levilactobacillus zymae*, *Limosilactobacillus pontis*, and *Pediococcus pentosaceus* [33, 36, 44]. In addition, yeasts such as *Saccharomyces*, *Pichia*, and *Rhodotorula* species are predominant microorganisms in the fermentation of *Borde* [45]. This microbial population interaction is vital in determining the nutritional value, safety, and sour taste of *Borde* [36].

#### 2.2.5. *Shamita*

**Figure 9:**
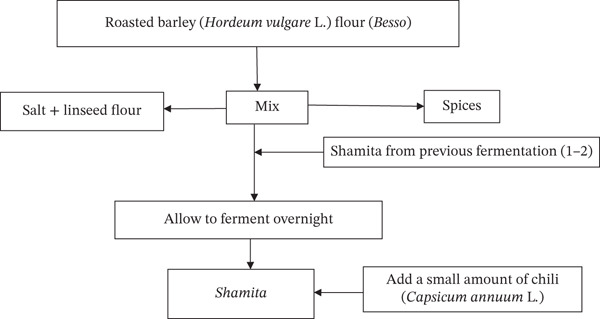
Flow chart of the *Shamita* production process [76].

It is primarily consumed in Western Ethiopia, specifically in the East Wollega Zone of the Oromia Region. *Shamita* is a traditional low‐alcohol (1.5%–0.5% *v*/*v*), sour, thick beverage that resembles a porridge fermented beverage [47]. Malt is not required for the saccharification process in the production of *Shamita*, similar to other traditional Ethiopian fermented beverages such as *Tella*, *Korefe*, and *Borde* [76]. *Shamita* is made by mixing roasted barley flour (*besso*), salt, linseed flour, cardamom flour, and a tiny bit of spice with water to produce a slurry liquid (Figure 9). One to two liters of *Shamita* from the previous fermentation (back slope) is sometimes used as a starter culture for the next batch. The mixture was then left to ferment overnight and prepared for consumption by adding a small amount of bird′s eye chili (*Capsicum annuum* L.) [47, 76]. The shelf life of *Shamita* appears to be 1–2 days at ambient temperature after it is ready for consumption [76].

The use of spices such as Ethiopian caraway (*Netch azmud*), false cardamom, and ground chili, which also contribute extra flavors and microbial diversity, has an important effect on *Shameta* fermentation [32]. It is also used as a healthy meal substitute and is mostly consumed by vulnerable groups, including children, pregnant women, the elderly, and people recovering from illness [76]. *Shamita* is a vital source of nutrition because it is high in protein, carbohydrates, minerals, and B vitamins. *Shamita* is a nourishing beverage and a representation of community in Ethiopian culture, and it is commonly consumed during fasting periods, social gatherings, informal social interactions, and daily meals. In addition to strengthening community ties and promoting social cohesion, its preparation and sharing aid in preserving indigenous knowledge and traditional fermentation techniques passed down through generations [48].

The microbiology of fermented *Shamita* is characterized by a diverse population of yeasts, LABs, and other bacteria that collaborate to impart flavor, texture, acidity, and nutritional content to fermented *Shamita* [49]. The common bacterial community includes *Lactobacillus*, *Leuconostoc*, *Pediococcus*, *Lactococcus*, *Streptococcus*, *Staphylococcus*, *Micrococcus*, and *Bacillus* species that produce acids, reduce pH, and inhibit the growth of spoilage bacteria [47, 48, 77]. Yeasts such as *Saccharomyces* and *Rhodotorula* dominate yeasts in fermented *Shamita* [49]. According to Sanz‐López et al. [44], the bacterial diversity of *Shamita* samples is dominated by LABs, such as *Lacticaseibacillus sharpeae*, *Lacticaseibacillus songhuajiangensis*, *Limosilactobacillus pontis*, *Paucilactobacillus vaccinostercus*, *Leuconostoc falkenbergense*, and *Levilactobacillus zymae*, along with various *Lactobacillus* species.

#### 2.2.6. *Korefe*

It is a foamy fermented beverage made by the Qimant and Korem people of the Amhara and Tigray regions of Ethiopia [1]. *Korefe* has a thick consistency, a bitter flavor, an earthy aroma, and a mildly alcoholic, slightly sour taste. The alcohol content of *Korefe* is typically low to moderate, falling between 2% and 4% (*v*/*v*) [1, 43]. *Korefe* is prepared using nonmalted roasted barley (*derekot*), nonmalted unroasted barley (*kita*), malted nonroasted barley (*bikil*), and *gesho* powder. Similar to other Ethiopian fermented beverages, the fermentation process is natural and spontaneous. Fermentation was initiated by the action of microorganisms naturally present in raw materials, utensils, and the surrounding environment [11].

**Figure 10:**
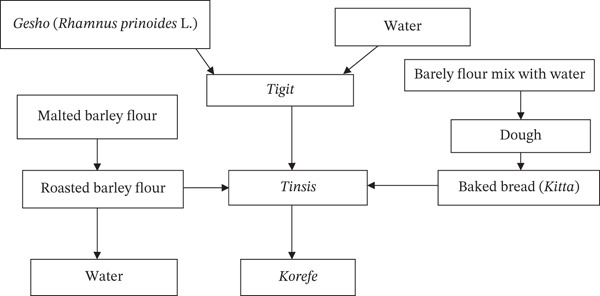
Flow chart of the *Korefe* fermentation process [11].

According to Getnet and Berhanu [11], the traditional *Korefe* fermentation process is divided into four basic stages, each characterized by the addition of ingredients at different times (Figure 10). In Stage I, *gesho* was mixed with water and left at ambient temperature in a local container (*gan*) for 72 h. This was the initiation stage for the extraction of flavor, aroma, bitterness, and antibiotics from *gesho* before real microbial fermentation began. In Stage II, *bikil* powder was added to water and fermented for 12 h. This was the first step in the real fermentation process and is commonly known as *tigit*. In Stage III, nonmalted barley bread (*kitta*) and water were added and fermented for 48 h. The semisolid mixture formed at this stage is known as *tinsis.* In Stage IV, roasted nonmalted barley powder (*derekot*) and water were added and fermented for 72 h. The semisolid mixture formed in this step is known as *liwes*. It is then fermented for 2–3 months, depending on environmental factors. *Korefe* and water (1:3 ratio) were mixed after the end of fermentation, and foam was formed. The formation of foam indicates that the beverage is ready for consumption. The shelf life of *Korefe* appears to be up to 3–7 days at ambient temperature [11, 78]. In the Qimant culture and nearby communities, *Korefe* has a significant social role and is brewed for group events such as social get‐togethers, religious holidays, harvest festivals, and events involving cooperative labor. As a sign of solidarity and teamwork, the beverage is frequently served in large clay pots or jars, from which participants share a drink [46].

The microbiology of the fermented beverage *Korefe* consists of a wide variety of yeasts, LABs, and other microorganisms, which are responsible for the quality of the beverage and the fermentation process. However, the predominant microorganisms that are present in the *Korefe* include yeasts and LABs, such as *Saccharomyces*, *Lactobacillus*, *Lactococcus*, *Pediococcus*, *Enterococcus*, and *Weissella*, in the fermented beverage *Korefe*. Furthermore, the genera Enterobacteriaceae, *Bacillus*, and *Micrococcus* are also present in the early stages of the process and are responsible for the degradation of the substrate [11].

#### 2.2.7. *Keribo*

Another popular fermented beverage in Ethiopia is *Keribo*, which is mainly made with roasted barley or wheat, sugar (honey), and bakery yeast. It is a nonalcoholic beverage (less than 1% *v*/*v*), and people who abstain from alcohol for health, cultural, or religious reasons or during fasting periods typically drink it. *Keribo* is a sweet‐and‐sour beverage that can be consumed as a light alcoholic beverage or as a soft drink [9, 79]. In recent years, *Keribo* has become particularly popular in the country during religious celebrations and social events, where heavy alcohol consumption is discouraged. It has different names in different places, including *Keneto*, *Gasilo*, *Mawudad*, *Filiteri* (from the word filter), and *Coca* (because it looks like Coca‐Cola). *Keribo* is a healthy and energetic beverage because it contains very little alcohol and trace amounts of vitamins, minerals, and barley‐derived carbohydrates [80].

**Figure 11:**
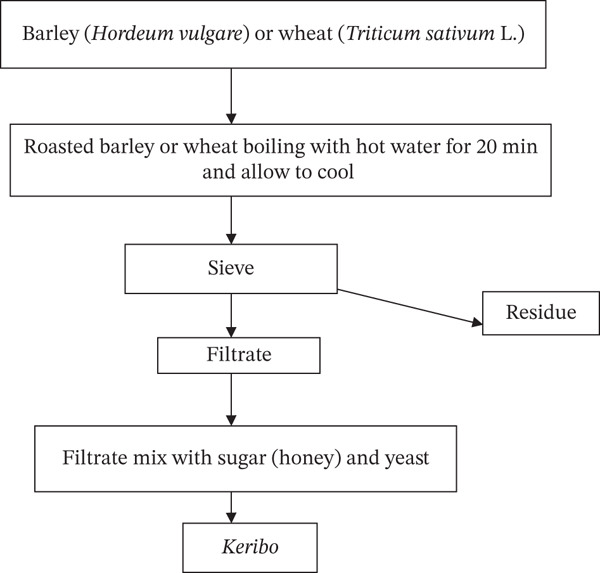
Flow chart of the traditional *Keribo* fermentation process [9].

According to Abawari [9], the first step in processing *Keribo* raw materials involves mixing roasted barley with hot water (Figure 11). After boiling for approximately 20 min, the mixture was filtered to remove the solid residue. The separated filtrate was then mixed with sugar (honey) and bakery yeast and allowed to ferment overnight. *Keribo* is then served to the consumer after additional sugar is added to the mixture. *Keribo* has a maximum shelf life of 2 days when stored at room temperature. It is frequently served as a nonalcoholic substitute for beverages such as *Tella* or *Tej* at social gatherings, religious ceremonies, and family get‐togethers. *Keribo*, a homemade, tasty substitute for commercial soft drinks, has become more popular in urban areas in recent years owing to its natural ingredients [9, 80].

The microbiological characteristics of fermented *Keribo* are dominated by the presence of LABs, AMBs, and yeasts that naturally initiate spontaneous fermentation in the product. Of the LABs, *Leuconostoc mesenteroides*, *Leuconostoc falkenbergense*, and *Liquorilactobacillus nagelii* are the most dominant in the *Keribo*, contributing to the development of acidity, flavor, and mild carbonation in the product through heterofermentative metabolism [9, 44]. Furthermore, AMBs are also present in the early stages of the process, responsible for the degradation of the substrate. Yeasts such as *S. cerevisiae* are predominant microorganisms in the fermentation of *Keribo*. This microbial population interaction is vital in determining the nonalcoholic nature, sweetness, safety, and sour taste of *Keribo* [9].

#### 2.2.8. *Ogol*

**Figure 12:**
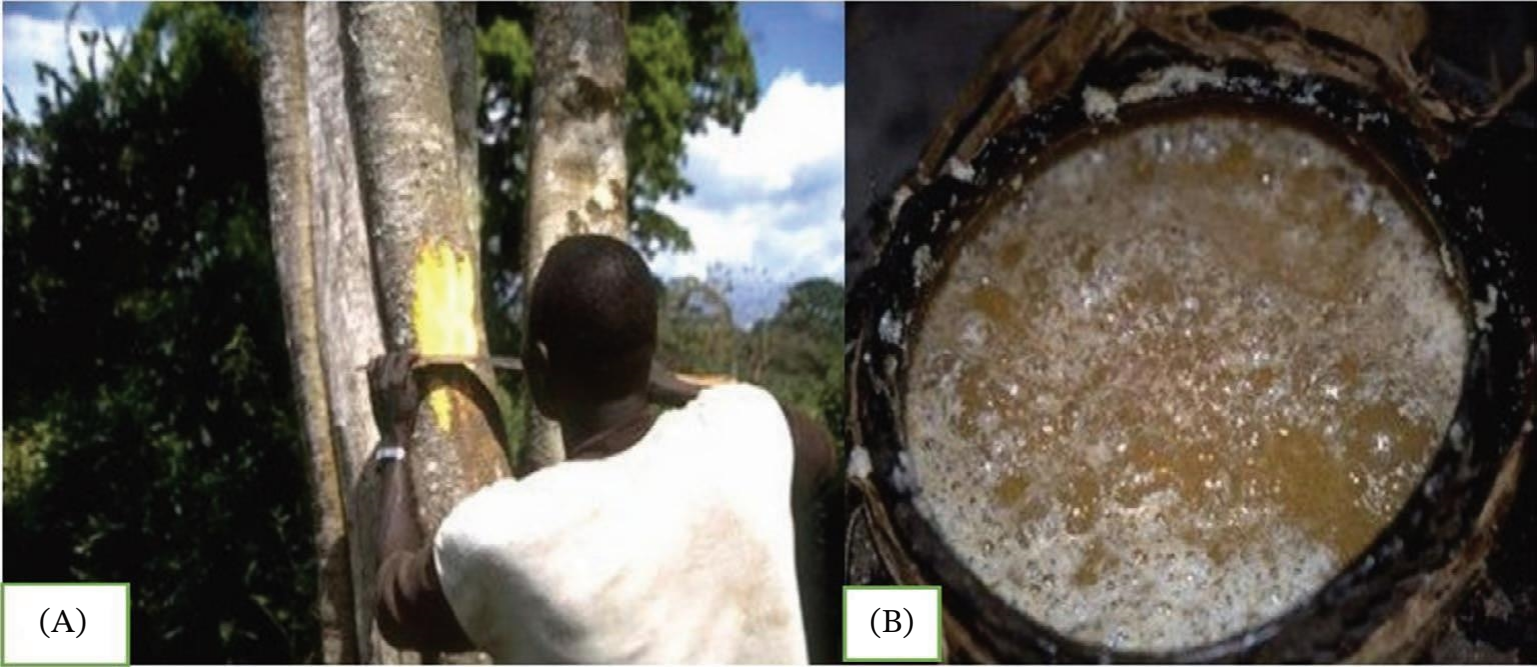
Traditional Ethiopian *Ogol* preparation. (A) The *Mange* (*Blighia unijugata* Bak) bark used for the production of *Ogol*. (B) *Ogol* fermented alcoholic beverage [7].

**Figure 13:**
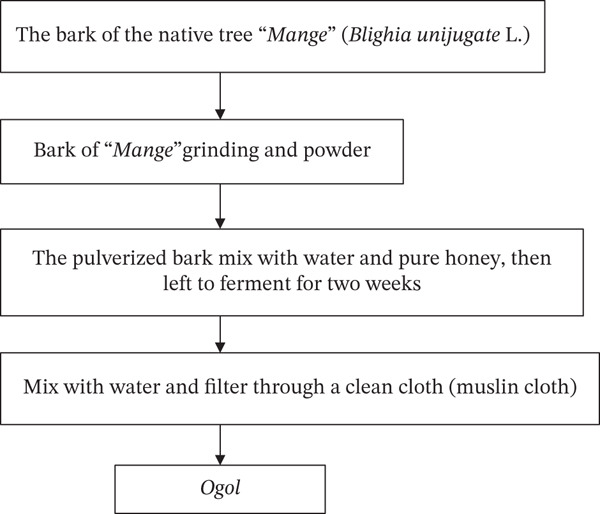
Flow chart of the *Ogol* fermentation process [7].

It is also known as *Ogol* wine or *Ogol Tej*, another traditional fermented honey wine beverage that is popular in southern Ethiopia, particularly among some communities that speak Omotic and Cushitic. Depending on the length of fermentation and the concentration of honey, its alcohol content typically varies from 6.5% to 17.5% *v*/*v* [7]. According to [7], *Ogol* preparation first, the bark of the native tree “*Mange*” (*Blighia unijugata* L.) is ground into a powder. For approximately 2 weeks, the pulverized bark, water, and wild honey are combined in a container and left to ferment spontaneously. Once the specified fermentation time has passed, a small amount of water is added, and the mixture is left to ferment anaerobically for a further 12–36 h (Figures 12 and 13). The shelf life of *Ogol* is 2–3 days at room temperature. It is then served to customers as *Ogol* after being filtered through a clean cloth. *Ogol*, which represents hospitality and celebration, is frequently prepared for important events like weddings, holidays, and neighborhood get‐togethers [78].

#### 2.2.9. *Cheka*

It is an indigenous cereal and vegetable‐based fermented beverage popular among the Konso, Gamo, and Dirashe communities in southwest Ethiopia. *Cheka* is made from cereals (primarily maize, sorghum, and finger millet) and vegetables (leaf cabbage, moringa, decne, and taro root) [42, 81]. It is a thick, porridge‐like beverage with alcohol content (2%–6% *v*/*v*) and a slightly sour taste [82].

**Figure 14:**
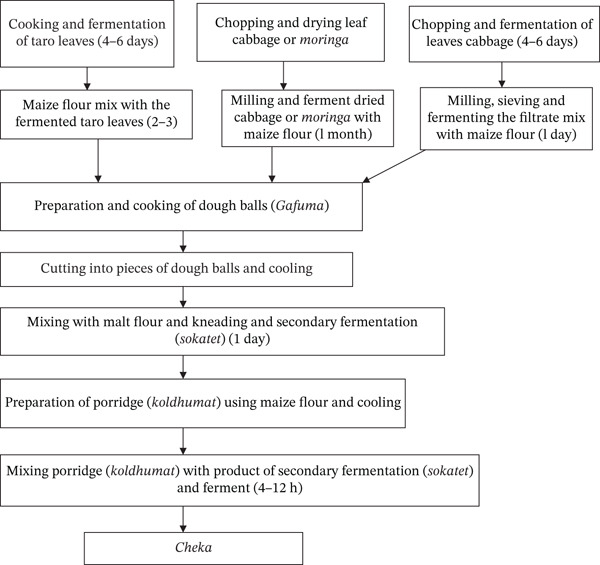
Flow chart of the traditional *Cheka* fermentation process [42].

According to Binitu [42], malting is the first step in the preparation of *Cheka*. Sorghum, maize, finger millet, and other cereals can be used alone or in combination to produce malts. The *Cheka* fermentation process relies on naturally present microorganisms in raw ingredients, equipment, and the environment. Chopped cabbage leaves and/or taro roots are anaerobically fermented for 4–6 days in a clean traditional container. The vegetable mixture is then fermented for a further 2–3 days after a small amount of maize flour is added. After being mashed and filtered, the fermented vegetable mixture was combined with fresh maize flour. The fermentation process was continued for an additional 12–24 h. Thereafter, water was added to the mixture, and it was fermented for a month. The fermented mixture is given the shape of a dough ball, which is locally known as *gafuma*. The mixture was then cooked at 93°C–95.5°C. Once the cooked *gafuma* has cooled, it is blended with the previously produced malt. The mixture was fermented for 12 h. This fermented mixture is locally called *sokatet*. At this stage, maize flour is used to make a very thick porridge known locally as *koldhumat*. The prepared porridge (*koldhumat*) is added to the vessel containing *sokatet* with enough water. Finally, the mixture is served to customers as *Cheka* after being allowed to ferment for an additional 4–12 h (Figure 14). Depending on environmental factors and sugar content, *Cheka* has a shelf life of 2–4 days [42].

*Cheka* provides energy and nourishment to local populations because it is rich in proteins, carbohydrates, dietary fiber, and vital micronutrients. *Cheka* is consumed by people of all ages, including infants, pregnant women, and lactating mothers [52]. Adult men typically consume up to 8 L of it daily, making it an inexpensive beverage and a significant contributor to the nutritional security of low‐income consumer groups [52, 82]. In addition to being a beverage, *Cheka* is a staple food with deep cultural significance that is shared during rituals, social gatherings, and community events. Additionally, they contribute to the local economy by generating household income and strengthening social bonds through shared production and consumption [82].

The microbiology of *Cheka* fermentation is characterized by a complex and dynamic community of microorganisms dominated mainly by LABs and yeast. The most dominant LABs in the *Keribo* are *Limosilactobacillus*, *Pediococcus*, and *Lactobacillus* genera. Among these LABs, *Limosilactobacillus panis*, *Limosilactobacillus fermentum*, *Pediococcus pentosaceus*, and *Lactobacillus delbrueckii* are predominant species in *Cheka* beverage [44, 53, 83]. Yeast species, such as *Saccharomyces*, *Candida*, and *Pichia*, are also found to be part of the microflora of *Cheka* beverage [53].

### 2.3. Nutritional Benefits of Traditional Fermented Beverages

TFABs such as *Borde*, *Korefe*, *Cheka*, and *Shamita* are a source of vital nutrients and energy because they are high in protein, carbohydrates, vitamins, and minerals from their cereal and grain ingredients (Table 1) [11, 35, 38, 39]. Despite being more alcoholic, *Tella*, *Areke*, and *Tej* contain antioxidants and micronutrients from barley, honey, and herbs during fermentation [84, 85]. Traditional fermentation improves nutrient content and digestibility by breaking down complex carbohydrates; adding vitamins (especially the B complex), amino acids, and organic acids; and introducing helpful microbes such as LABs and yeasts. For low‐income communities with limited access to a wide range of foods, TFABs are an important source of energy, protein, and minerals [10, 36]. In addition to their nutritional benefits, TFABs promote mental health and social cohesiveness. *Borde*, *Booka*, *Ogol*, *Cheka*, *Korefe*, and *Shamita* can help prevent malnutrition by offering essential calories and protein, especially in children, workers, nursing mothers, the elderly, and those recuperating from illness [36, 85].

The nutritional content of high‐alcoholic fermented beverages such as *Cheka*, *Korefe*, *Borde*, and *Shamita* is higher than that of highly alcoholic drinks such as *Tej*, *Areke*, and *Tella*. The study by Binitu et al. [52] revealed that the total ash, crude fat, crude protein, and carbohydrate contents of the *Cheka* samples ranged from 3.12 to 4.44 g/100 g, 1.17 to 1.81 g/100 g, 0.65 to 0.93 g/100 g, and 14.16 to 19.03 g/100 g, respectively. The ash, fat, soluble protein, total protein, and carbohydrate content of *Borde* were 9.55%, 3.31%, 6.88%, 3.66%, and 8.88%, respectively. *Shamita* had more total protein, fat, and ash values of 10.37%, 3.46%, and 6.85%, respectively [10, 36]. The higher levels of protein and carbohydrates in *Cheka*, *Borde*, and *Shamita* justify the assertion that they can be utilized as meal replacements. It is also used for medical and ritual purposes in some cultures.

TFABs are rich in probiotics, which provide health benefits, including antimicrobial and antioxidant properties [86]. Probiotics have recently received significant attention owing to overwhelming evidence of their positive health effects [87]. The consumption of these traditional beverages offers considerable health advantages to communities and contributes to economic benefits for society [88]. According to Narayan et al. [88], probiotics hold great promise for the treatment and prevention of many types of diseases, both infectious and noninfectious. Probiotics have been proven to have antimicrobial activity, improve lactose metabolism, lower serum cholesterol, prevent gastrointestinal infections, strengthen the immune system, and have antidiarrheal, anticarcinogenic, antimutagenic, and inflammatory bowel disease–relieving properties [89].

### 2.4. Microbial Role Toward Nutritional and Safety Value

A variety of microorganisms found in TFABs are employed as probiotics to improve the sensory qualities of drinks, bacteriocins, organic acids, and hydrogen peroxide, which enable them to function as efficient biopreservatives and develop nutraceuticals for the production of functional drinks with higher nutrient bioavailability [71, 84, 90]. In many communities worldwide, there is a traditional belief that certain fermented foods and beverages possess therapeutic properties. Hence, it is known that rural communities consume fermented food and beverages like *Borde*, *Shamita*, *Korefe*, *and Cheka*, which have health benefits [8, 84]. LABs and yeasts are among the many probiotic microorganisms present in TFABs and play an essential role in fermentation, flavor development, and potential health benefits [84, 91]. Nisin, acidophilin, lactocidin, brevicin, helveticin, and other bacteriocins are produced by LABs [92–94]. LAB includes the following genera: *Leuconostoc*, *Enterococcus*, *Lactobacillus*, *Bifidobacterium*, and *Streptococcus* [84]. According to Mulaw et al. [91], *Lactobacillus plantarum* strain CIP103151, *Lactobacillus paracasei* strain NBRC 15889, and *Lactobacillus plantarum* strain JCM 1149 found in fermented *Borde* and *Shamita* provide antibacterial qualities that protect the gastrointestinal system from a variety of foodborne infections [33].

Traditional fermentation occurs spontaneously as a result of environmental microbes and microorganisms associated with the source raw material [71, 95, 96]. This microbial activity caused biochemical changes that led to acidification and increased the shelf life of conventional alcoholic beverages. Fermentation of lactic acid lowers pH and inhibits bacterial growth and spoiling. Blandino et al. [97] reported that lactic acid fermentation contributes to the preservation of beverages, and it is due to hydrogen peroxide production and organic acids by the LABs. The ability of LABs to convert carbohydrates into organic acids (butyric, propionic, lactic, and acetic acids) lowers the product′s pH to a point where spoiling and/or pathogens cannot survive [98]. The number of some of the most common microorganisms, including bacteria from the genera *Bacillus*, *Lactobacillus*, *Staphylococcus*, *Micrococcus*, and *Enterobacteriaceae*, and yeasts from the genus *Saccharomyces*, significantly increased in a study by [1] on the microbiology of fermentation in *Borde* and *Shamita*. Additionally, the pH of *Shamita* decreased from 5.82 to 3.98, and the pH of *Borde* dropped from 5.2 to 3.6.

Fermentation improves the flavor and aroma of beverages, making them more appetizing. Owing to their organoleptic qualities, fermented beverages are more widely accepted by consumers than unfermented beverages [15]. TFABs depend on microorganisms (LAB and yeast), and the byproducts of their metabolism provide the fermenting material with its unique flavor and aroma, in addition to providing acidity [64]. Numerous organic acids and volatile chemicals are produced during grain fermentation, which contribute to the diverse flavor combinations of the products. These fermented drinks have high concentrations of lactic acid [99, 100].

## 3. Challenges of Traditional Ethiopian Fermented Beverages

Numerous obstacles limit the quality, safety, and commercial potential of TFABs (*Tella*, *Tej*, *Borde*, *Korefe*, *Keribo*, *Cheka*, and *Shamita*). The lack of standardization in the production of TFABs is one of the main challenges because these beverages are usually made at the household level without controlled fermentation processes, resulting in variations in their quality, safety, and consistency [28, 29]. TFABs face safety issues due to uncontrolled production processes, inconsistent fermentation techniques, and inadequate quality control [19]. Traditional fermented drinks are produced at home without adhering to strict guidelines for sanitation, fermentation control, or raw material handling [21]. Containers for fermentation and storage are usually made of materials that are difficult to clean or that could contaminate the beverages. Additionally, the preparation of water might not be potable, which increases the risk of contamination [30]. TFABs have several drawbacks that prevent them from being widely marketed. These include insufficient knowledge of proper packaging materials, lack of institutional support systems, lack of market networking, lack of access to processing tools, lack of quality assurance, and lack of training, just a few of these issues [31, 101].

Hygiene and quality control are vital for the successful commercialization of traditional beverages [102]. Poor hygiene and posthandling fermentation (poorly dried, not pasteurized, or not refrigerated) are partially associated with the present low technical fermentation technique, which can shorten the shelf life of products and make them susceptible to contamination [103]. Similarly, many small‐scale manufacturers use locally accessible materials to package their fermented products, which, while acceptable for local consumption, are often unfavorable for prolonging shelf life or being attractive at the point of sale. However, household processors face various institutional and technical barriers, such as inadequate government regulations that encourage and facilitate small‐scale fermentation processing; inadequate supply of raw materials; poor infrastructure, which is often seen in impoverished rural areas; and restricted access to outside inputs and technical assistance [102].

Many types of Ethiopian TFABs have a short shelf life because of poor preservation and packaging methods, which makes their distribution, storage, and transportation challenging [19, 30, 31]. The informal and unregulated production of traditional beverages is influenced by the lack of appropriate regulatory frameworks and quality assurance policies. Furthermore, TFAB production and marketing are still mostly informal, without government assistance, financial investment, or technological advancement, which limits their commercialization and economic influence [30]. The traditional fermentation knowledge that is typically passed down orally from generation to generation is being undermined by modernization and urbanization [15].

Backslopping is not a common practice among Ethiopian TFABs, where a small quantity of the previous successful fermentation batch is used as a starter culture for the next batch [30, 78]. Backslopping is effective and economical and allows the producers of TFABs to produce continuously while saving time and resources [104, 105]. However, there are several possible problems when backslopping is used frequently [106]. Microbial contamination is a significant issue because, over time, backslopped cultures may acquire undesirable microorganisms, such as pathogens or spoilage bacteria, which can lead to souring, off‐flavors, or even potentially harmful products. Repeated backslopping also favors fast‐growing microbes over slower growing beneficial yeasts and LABs, which reduces microbial diversity and affects the taste, aroma, and fermentation ability of the final product. Another challenge is the loss of desirable strains due to variations in microbial populations, which leads to variations in alcohol content and can impact product quality and market acceptability [90, 107]. The shelf life of backslopping is shortened, which causes microbial imbalances that could speed up the spoilage of the beverages. Finally, the natural characteristic flavors of the final beverages may be lost over time because of the continued metabolic activity of the local microbial diversity [106–108].

Public policy should guarantee that efficient systems are established to control and promote responsible alcohol use and safeguard people who might be negatively impacted by it, either directly or indirectly. This could manifest as social norms and rules or be more formalized, such as legislation, and will differ throughout countries and cultures. The minimum drinking age is one of many examples of current public laws and educational initiatives aimed at preventing abuse, which can be undertaken when under the influence of alcohol (operating machinery, driving a vehicle, etc.), restricting sales through taxation or more specifically at certain periods of time or events, and improving knowledge of the potential effects via warning labels and consumer awareness campaigns [109, 110].

### 3.1. Health Implications and Safety Challenges of Traditional Fermented Beverages

The production of alcoholic beverages by a mixed microbial consortium via traditional fermentation can result in the synthesis of mixed alcohols that form methanol and other volatile congeners [111]. Noncommercial alcoholic drinks are potentially associated with social, health, and financial issues [3]. However, because illegal drinks contain contaminants and have unpredictable alcohol levels, people who choose noncommercial alcohol face greater risks. It should be noted that excessive consumption of noncommercial beverages can result in numerous difficulties [21, 111]. The first issue with noncommercial beverages is that there are no set criteria for their manufacture, and some fail to meet acceptable content and hygiene requirements. Second, in an attempt to boost the alcohol content of distilled drinks and enable greater revenue, certain manufacturers have been reported to add surrogates (e.g., charcoal, methanol, and car battery acid) [64]. Aliphatic alcohols that are harmful to liver cells (hepatotoxic) are frequently found in homemade beverages, and their concentrations vary greatly based on the production process and raw ingredients utilized [111]. Thus, for example, it is assumed that high liver cirrhosis mortality rates are caused by a heavy intake of homemade alcohol. According to a chemical examination of samples of homemade beverages, 82% contained methanol, 94% contained 2‐butanol, and 100% contained 1‐propanol, isobutanol, and isoamyl alcohol [112].

Many *Tella* and other TFAB samples collected from Addis Ababa, Gojam, and North Shoa in Ethiopia were analyzed for their ethanol, methanol, and fusel oil contents by [69]. The mean values of methanol, fusel oil, and ethanol were 35 ppm, 66 ppm, and 3.6%, respectively. Isobutanol, 1‐butanol, 2‐butanol, and 1‐propanol, which have higher alcohol levels, can all be referred to as fusel oil or fuselol [111]. A small amount of fusel oil contributes to the good flavor of the product. However, fusel oil is unhealthy if consumed in quantities greater than 1000 g/hL of pure alcohol [113].

The increased methanol concentration in TFABs has detrimental effects on health [64]. Methanol is usually formed by spontaneous, natural, and uncontrolled fermentation [71]. The methanol content of *Tella* and *Cheka* is much lower than the European Union (EU) maximum limit (EEC No 1576/89). The concentration of fluoride ions in TFABs is another significant food safety concern, due to the naturally occurring fluoride in the water source of the Great Rift Valley, Ethiopia [114]. The concentration of fluoride ions was higher in traditional drinks gathered close to the Rift Valley [115]. In contrast to the victims′ blood methanol levels of 1500–2000 mg/L, laboratory examinations conducted by WHO and NAFDAC (National Agency for Food, Drug Administration and Control) indicated that the beverage contained 16.3% methanol. Methanol poisoning symptoms in victims include headache, nausea, vomiting, dizziness, weakness, respiratory problems, blurred vision, blindness, weight loss, and stomach aches [116].

Local drinks are typically made in households, usually in unsanitary conditions, and thus are prone to contamination by microflora. Humans, sewage, raw materials, utensils, processing equipment, the environment, improper handling and storage conditions, and rodents are the primary sources of contamination [117, 118]. *Staphylococcus aureus*, *Escherichia coli*, *Bacillus* species, *Streptococcus* species, *Proteus* species, *Rhizopus stolonifer*, *Aspergillus flavus*, and *Aspergillus niger* are a few pathogenic microorganisms that can contaminate beverages [117–119]. These pathogenic microbes have the potential to be dangerous to humans, present a major risk to consumer health, and cause financial losses to manufacturers [118, 120]. According to reports, the main issue with conventional alcoholic beverages is their short shelf life or stability. Conventional fermentation takes a long time, the brewed products degrade easily, and if they are not sold or consumed immediately, they result in financial loss. Small amounts are brewed, while there are numerous consumers [60].

Microbiological quality analysis of *Keribo* from Jimma zone homes and open markets revealed the presence of pathogenic microorganisms and hygienic indicators, which have been identified in many samples of *Keribo.* The mean counts of aerobic spore formers (ASFs), AMBs, aerobic LABs, and yeasts were 4.96 ± 2.80, 2.34 ± 2.37, 2.70 ± 2.07, and 4.96 ± 0.60 log CFU/mL, respectively. The yeast count at 24 h of fermentation ranged from 10^7^ to 10^8^ CFU/mL, whereas the Enterobacteriaceae levels were approximately 10^6^ CFU/mL. Mesophilic bacterial counts of approximately 10^9^ CFU/mL and yeast counts of 10^5^–10^7^ CFU/mL were detected in freshly made *Borde*. *Borde* becomes unsafe for consumption after 12 h of storage at room temperature because of these high microbe counts [36].

Mycotoxin levels in conventional beverages are highly significant for public health concerns. Both acute and chronic mycotoxicosis are associated with these diseases [68]. The study assessed the aflatoxin content of two Ethiopian traditional alcoholic beverages (*Tella* and *Areke*) collected from three Gojam zonal administrative towns, which are located in the East, West, and Awi zones, namely, in Enjibarra, Finoteselam, and Debremarkos towns, respectively. The findings showed that the quantities of aflatoxins found in the *Areke* samples had vague chromatogram peaks, which may have been “masked/modified” aflatoxins. While in Debremarkos, Fintoselam, and Enjibarra, the mean total aflatoxin levels were 12.8 ± 4.43, 14.4 ± 8.76, and 11.4 ± 3.38 * μ*g/kg, respectively [63]. Mycotoxins, such as aflatoxins and fumonisins, are produced by *Aspergillus*, *Fusarium*, and *Penicillium*. Aflatoxin B1 (AFB1) has teratogenic, mutagenic, carcinogenic, and toxic properties [121].

TFABs play a significant role not only in the nutritional context but also as functional foods with potential health benefits in the prevention and management of communicable and noncommunicable diseases [88, 89]. Fermented alcoholic beverages are naturally rich in beneficial microorganisms, such as LABs and yeasts, which help promote a better balance of gut microbiota and boost the immune system [122]. The presence of probiotics in fermented drinks can inhibit communicable diseases through the production of organic acids, bacteriocins, and antimicrobial metabolites, thereby reducing the incidence of gastrointestinal and diarrheal diseases. In addition, traditional alcoholic fermented beverages enhance the bioavailability of nutrients and produce bioactive compounds, such as vitamins, peptides, and antioxidants, which strengthen the body′s resistance to communicable diseases [123]. Regular consumption of TFABs has been linked to beneficial effects against diseases such as cardiovascular diseases, hypertension, Type 2 diabetes, and various cancers. These positive effects are linked to their capacity to modulate lipid profiles, increase glucose metabolism, decrease oxidative stress, and reduce inflammation [26, 124].

## 4. Support Services to Promote Traditional Fermented Beverages

Ethiopian TFABs have the potential to grow if they have access to sufficient support services, including financial services, technical training, public policy, marketing assistance, business skills development, and modern fermentation technology [30, 31, 125, 126]. Scientists and decision‐makers are increasingly aware of the importance of traditional fermentation processes as a component of food security plans [10, 110]. TFABs were developed largely as an art rather than through scientific principles. They are affordable and incorporated into the village culture and frequently use locally accessible raw materials. However, the preparation of traditionally fermented beverages lacks a scientific basis [127]. Local beverage products are marketed using illegal marketing strategies. *Tella*, *Tej*, and *Areke* are examples of beverages with a high alcoholic content that provide an issue due to the health risks associated with excessive consumption [111]. Thus, control of production and supervision with the development of a comprehensive national alcohol policy is required.

There should also be a strong linkage among the different institutions involved in beverage development in the country, and a networking structure needs to be established to exchange information on national research priorities, best practices, and the adoption of technologies and advances in beverage development. It is also recommended that the full potential of beverage development should be supported by strong policies [125]. The development, promotion, and regulation of the beverage industry depend on the establishment of a coordination framework through public–private sector collaborations, such as a national board [31]. Small‐scale farmers can readily take advantage of the numerous opportunities offered by local markets located on their doorsteps and in nearby villages. However, beyond the local and village levels, the fermented beverage market potential needs to deal with a number of prerequisites, such as larger production facilities, more ad hoc equipment, consistency and dependability in quality control, labeling, and licensing [103]. Due to regional variations in customer preferences, market accessibility, and transportation infrastructure, effective marketing methods may vary. Depending on whether fermented products have a long shelf life, they require various strategies. However, regardless of the marketing strategy used locally, quality control methods are crucial for producing safe and sanitary products that help small‐scale fermentation succeed [31].

The following are some possible policy‐level government initiatives to encourage fermentation activities: implementing livelihood support policies that enable rural and urban beverage processors to adopt diverse and sustainable livelihood strategies by offering cross‐sectoral support to the natural resources, agriculture, and development sectors. Establishing specific regulations to offer unambiguous assistance and direction on how to enhance and maintain the quality, safety, and standards of traditional beverage processing is essential. Establishing regulations to assist traditional producers in promoting the sale of their fermented drinks through branding and other means is essential. Legislation should be developed to prevent irresponsible consumption of alcohol. While policy support for the traditional beverage sector is required for livelihood‐based enterprise diversification, regulation is necessary to safeguard those who may be negatively impacted by it, either indirectly or directly [31, 34, 125].

Household‐level fermentation processes in Ethiopia have been developed as an art rather than using scientific principles; therefore, the tools and procedures involved are fairly simple, although the microbiological and biochemical aspects of several of these processes are intricate and not well understood [127]. Brewers use their judgment to determine the fermentation period. Physical factors such as temperature, relative humidity, and the degree of agitation and aeration are frequently poorly managed, and production methods are not standardized in household fermentation. There is no way to ensure that an uncontaminated fermentation environment, unpredictable processing environment, facilities, equipment, and handlers′ hygiene are not evaluated [31].

Training on proper hygiene techniques that avoid contamination and how to increase fermentation efficiency is especially necessary to produce consistently acceptable outputs in terms of both quantity and quality [102]. Enhancing shelf life may also be achievable through training in suitable technologies, such as more effective drying, pasteurization, vacuum filtration, and/or refrigeration, which prevent fermentation and hence extend the product′s shelf life [103]. Traditional fermented beverages are made using age‐old methods and readily available ingredients [36]. Household producers are frequently limited in their options for fermented beverage packaging by what is readily accessible in their area, which may include glass bottles and clay pots. An increased understanding of proper fermentation product packaging using better materials rather than primitive equipment would further increase the product′s shelf life and marketability [31].

Many entrepreneurs lack sufficient experience in managing their businesses. With the opening of markets, the level of competition for entrepreneurs has increased significantly, while the expectations and needs of customers have grown. For businesses operating in an open market to succeed in the long run, assistance in the form of training and situation‐specific guidance on company management issues is frequently essential. Training in processing methods and quality management is equally important [101]. Successful fermentation activities can be significantly impacted by education, which is acknowledged as a key component influencing people′s ability to engage in income‐generating activities. Even for trading at the local level, basic bookkeeping and accountancy abilities are frequently needed, and manufacturers of fermented goods benefit from personal traits, including self‐assurance, the capacity to try new things and take calculated risks, and attention to detail. To grow their fermentation activities beyond local trade into a small business, traditional entrepreneurs need to have more advanced entrepreneurial abilities. Such skills may include bookkeeping, administration and planning, management of supplies of fermentation materials and equipment and starter cultures and fermentation substrate, management of packaging requirements, meeting legal requirements, logistical coordination of transport and distribution, and negotiation skills and marketing [31, 34, 110].

Minimal financial resources are required to carry out household beverage fermentation for local trade. The importance of financial resources will increase as an organization grows in size and produces products that can be consistently exchanged for revenue, thus requiring access to technical assistance and improved techniques to function more effectively [101]. For example, increased technological equipment, information sharing, and exchange visits can be facilitated by internal or external funding and provide training to expand cultivation skills. The types of credit that are accessible vary between countries, but central and local governments, some private groups, and cooperatives are typically excellent places to start new businesses [30]. The lack of suitable starter cultures, process controls, and basic scientific knowledge of the procedures is a frequent barrier to the effective transfer and adaptation of technology. It is crucial to support research and development aimed at improving the knowledge of the technologies used in traditional household fermentations by strengthening institutional capabilities in developing countries [31, 125].

## 5. Opportunities and Prospects of Traditional Fermented Beverages

TFABs have great potential to enhance dietary health, preserve cultural heritage, and promote economic development [3, 30]. They have great potential for value addition, branding, and export marketing because of their unique sensory attributes and cultural roots. TFABs can attract niche markets interested in naturally fermented products high in probiotics, as the demand for genuine, artisanal, and health‐conscious traditional beverage products grows worldwide [30]. The future of these traditional Ethiopian beverages depends on integrating traditional knowledge with modern biotechnological advancements, encouraging value addition, and investigating the probiotic and functional properties of these beverages [31].

Technology transfer, skill development, and innovation can be encouraged by building research partnerships among local producers, agricultural extension services, food research institutes, and universities [31, 126]. These partnerships can also improve consistency, safety, and fermentation process control and optimization, thereby enabling scalable and long‐term commercialization. To meet the needs of regional producers and entrepreneurs involved in the value chains of TFABs, universities, food research institutes, and agricultural extension services may offer comprehensive training programs, workshops, and extension services [126]. Collaborations are crucial for technical innovation because they enable local producers to use modern machinery and promote advancements in fields such as packaging, distribution, and quality control [128, 129]. Furthermore, Ethiopia′s rich fermentation heritage can be promoted internationally by promoting these beverages through tourism, cultural events, and export‐focused branding. The development of globally recognized products that blend cultural authenticity with contemporary quality standards can be ensured by government support, investment incentives, and certification programs [102].

Standardized starter cultures should be introduced to ensure consistency, safety, and better product quality [129]. Additionally, producers should be trained in hygiene and quality control, and fermentation techniques should be improved through scientific research. TFABs must be produced under strict hygienic standards to guarantee product consistency and safety [30]. Microbial contamination can be considerably decreased by implementing training programs for regional producers on safe ingredient handling, controlled fermentation, and sanitation [31]. Product quality and marketability can be further improved by establishing small‐scale processing industries and fortifying the regulatory frameworks. The development of locally adaptable, low‐cost packaging and preservation technologies can enhance marketability, increase distribution potential, and reduce spoilage [103]. Using digital marketing tools, traditional festivals, and ecotourism to promote these TFABs will open up new business opportunities for rural and semiurban residents [130, 131].

The identification of metabolic pathways, functional capabilities, and microbial diversity in TFABs has become dependent on continuous innovation through omics‐driven research [132]. Using omics‐driven research, a range of native strains is first identified through microbial bioprospecting, and beneficial genes linked to probiotic traits, ethanol production, enzyme synthesis, detoxifying properties, and safety markers are identified through whole‐genome sequencing [133]. Researchers can help standardize and commercialize TFABs, improve fermentation processes, enhance product safety and consistency, and make it possible to employ promising microbial strains in the industry by incorporating omics technology into TFABs [134]. In general, Ethiopian TFAB commercialization can increase food and nutrition security while also providing economic benefits. To accomplish this commercialization strategy, starter cultures should be defined to improve consistency, optimize fermentation processes, purchase appropriate equipment, establish a regulatory pathway, prioritize sustainability, and inform consumers of the economic value of TFABs [30, 40].

## 6. Conclusions

Ethiopian TFABs have played important social, religious, nutritional, and therapeutic roles in Ethiopia for centuries. These beverages offer vital nutrients and bioactive compounds that support nutrition and public health, in addition to reflecting Ethiopia′s rich heritage of culture and traditional knowledge. The production of TFABs is still mostly informal and unstandardized, leading to problems with quality assurance, safety, and commercialization, despite their significant cultural and nutritional benefits. Traditional knowledge systems and the continuity of production have been further threatened by urbanization and industrialization in recent years. Several issues, such as a lack of standardization, poor hygiene, and a lack of institutional support, continue to limit the quality, safety, and commercialization of TFABs in Ethiopia. Their shelf life and market potential are further limited by informal production methods, low technical capability, and inadequate packaging and preservation techniques. Ethiopian TFABs can be standardized and commercialized by employing starter cultures, improving fermentation techniques, introducing technological innovations, creating a regulatory framework, and informing customers about the benefits of these beverages.

## Funding

This study was funded by Debre Markos University, 10.13039/501100021567.

## Conflicts of Interest

The author declares no conflicts of interest.

## Data Availability

Data sharing is not applicable to this article as no datasets were generated or analyzed during the current study.

## References

[1] Wedajo L. B. , Microbiology of Ethiopian Traditionally Fermented Beverages and Condiments, International Journal of Microbiology. (2020) 2020, no. 1, 1478536, 10.1155/2020/1478536, 32148508. 32148508 PMC7042527

[2] Kohajdova Z. and Karovicova J. , Fermentation of Cereals for Specific Purpose, Journal of Food and Nutrition Research. (2007) 46, no. 2, 51–57.

[3] World Health Organisation , Global Status Report on Alcohol and Health, 2014, World Health Organisation, https://www.who.int/publications/i/item/global-status-report-on-alcohol-and-health-2014.

[4] Kabak B. and Dobson A. D. W. , An Introduction to the Traditional Fermented Foods and Beverages of Turkey, Critical Reviews in Food Science and Nutrition. (2011) 51, 248–260, 10.1080/10408390903569640, 2-s2.0-79952386824. 21390945

[5] Tekle B. , Anuradha J. S. , Fantaw D. , Gebreslassie T. , Mohan M. R. , Baraki H. , and Gebregziabher K. , An Insight Into the Ethiopian Traditional Alcoholic Beverage: Tella Processing, Fermentation Kinetics, Microbial Profiling and Nutrient Analysis, LWT. (2019) 107, 9–15, 10.1016/j.lwt.2019.02.080, 2-s2.0-85062439074.

[6] Kemal S. and Koricha A. D. , The Art, Microbial Quality, Safety, and Physicochemical Characteristics of Jikita: A Traditional Ethiopian Fermented Beverage, International Journal of Food Science. (2024) 2024, 6698831, 10.1155/2024/6698831, 39044801. 39044801 PMC11265947

[7] Teramoto Y. , Sato R. , and Ueda S. , Characteristics of Fermentation Yeast Isolated From Traditional Ethiopian Honey Wine, Ogol, African Journal of Biotechnology. (2005) 4, no. 2, 160–163, 10.4314/ajb.v4i2.15072.

[8] Elema T. B. , Olana B. N. , Elema A. B. , and Gemeda H. F. , Processing Methods, Physical Properties and Proximate Analysis of Fermented Beverage of Honey Wine Booka in Gujii, Ethiopia, Journal of Nutrition & Food Sciences. (2018) 8, no. 669, 10.4172/2155-9600.1000669.

[9] Abawari R. A. , Microbiology of *Keribo* Fermentation: An Ethiopian Traditional Fermented Beverage, Pakistan Journal of Biological Sciences. (2013) 16, no. 20, 1113–1121, 10.3923/pjbs.2013.1113.1121, 2-s2.0-84876431007, 24506010. 24506010

[10] Tafere G. , A Review on Traditional Fermented Beverages of Ethiopian, Journal of Natural Sciences Research. (2015) 5, no. 15, 94–103.

[11] Getnet B. and Berhanu A. , Microbial Dynamics, Roles and Physico-Chemical Properties of Korefe, A Traditional Fermented Ethiopian Beverage, Biotechnology Society. (2017) 9, no. 7, 156–175.

[12] Bacha K. , Mchari T. , and Ashenafi M. , The Microbial Dynamics of *′borde′* Fermentation, A Traditional Ethiopian Fermented Beverage, SINET: Ethiopian Journal of Science. (1998) 21, no. 2, 195–205, 10.4314/sinet.v21i2.18120.

[13] Weldeabzgi S. G. , Sbhatu D. B. , Berhe G. G. , and Gebreyohannes G. , Optimization of Fermentation Parameters and Characterization of Finger Millet-Based Suwa: A Traditional Ethiopian Ale, Scientific African. (2025) 29, e02905, 10.1016/j.sciaf.2025.e02905.

[14] Nemo R. and Bacha K. , Microbial, Physicochemical and Proximate Analysis of Selected Ethiopian Traditional Fermented Beverages, LWT. (2020) 131, 109713, 10.1016/j.lwt.2020.109713.

[15] Paulos M. M. , Gqaleni N. , and Chelule P. K. , Méndez-Vilas A. , Advantages of Traditional Lactic Acid Bacteria Fermentation of Food in Africa, Current Research, Technology and Education Topics in Applied Microbiology and Microbial Biotechnology, 2010, Formatex, 1160–1167.

[16] Shumye T. and Admassu S. , Characterization of Borde: A Traditional Ethiopian Cereal-Based Fermented Beverage - Microbial Analysis, Rheological Profiling, and Functional Attributes, Journal of Agriculture and Food Research. (2024) 18, 101498, 10.1016/j.jafr.2024.101498.

[17] Jeyaram K. , Anand S. T. , Romi W. , Ranjita A. D. , Mohendro W. S. , Dayanidhi H. , Rajmuhon N. S. , and Tamang J. P. , Traditional Fermented Foods of Manipur, Indian Journal of Traditional Knowledge. (2009) 8, no. 1, 115–121.

[18] Hall C. M. and Sharples L. , Food and Wine Festivals and Events Around the World: Development, 2008, Management and Markets.

[19] Hotessa N. and Robe J. , Ethiopian Indigenous Traditional Fermented Beverage: The Role of the Microorganisms Toward Nutritional and Safety Value of Fermented Beverage, International Journal of Microbiology. (2020) 2020, no. 1, 891259, 10.1155/2020/8891259, 33488731. PMC7803167 33488731

[20] Hidalgo-Fuentes B. , de Jesús-José E. , Cabrera-Hidalgo A. D. J. , Sandoval-Castilla O. , Espinosa-Solares T. , González-Reza R. M. , Zambrano-Zaragoza M. L. , Liceaga A. M. , and Aguilar-Toalá J. E. , Plant-Based Fermented Beverages: Nutritional Composition, Sensory Properties, and Health Benefits, Foods. (2024) 13, no. 6, 10.3390/foods13060844, 38540834. PMC10969568 38540834

[21] Alemu T. T. and Kuyu C. G. , A Review of the Production, Quality, and Safety of Traditionally Fermented Cereal-Based Alcoholic Beverages in Ethiopia, Food Science & Nutrition. (2024) 12, no. 5, 3125–3136, 10.1002/fsn3.4012, 38726402. 38726402 PMC11077225

[22] Banwo K. , Oyeyipo A. , Mishra L. , Sarkar D. , and Shetty K. , Improving Phenolic Bioactive-Linked Functional Qualities of Traditional Cereal-Based Fermented Food (Ogi) of Nigeria Using Compatible Food Synergies With Underutilized Edible Plants, NFS Journal. (2022) 27, 1–12, 10.1016/j.nfs.2022.03.001.

[23] Samtiya M. , Aluko R. E. , Puniya A. K. , and Dhewa T. , Enhancing Micronutrients Bioavailability Through Fermentation of Plant-Based Foods: A Concise Review, Fermentation. (2021) 7, no. 2, 10.3390/fermentation7020063.

[24] Adebo A. O. , African Sorghum-Based Fermented Foods: Past, Current and Future Prospects, Nutrients. (2020) 12, no. 4, 10.3390/nu12041111, 32316319. PMC7231209 32316319

[25] Degrain A. , Manhivi V. , Remize F. , Garcia C. , and Sivakumar D. , Effect of Lactic Acid Fermentation on Color, Phenolic Compounds and Antioxidant Activity in African Nightshade, Microorganisms. (2020) 8, no. 9, 10.3390/microorganisms8091324, 32872680. PMC7564239 32872680

[26] Cuamatzin-Garcia L. , Rodriguez-Rugarcia P. , El-Kassis E. G. , Galicia G. , Meza-Jimenez M. L. , Banos-Lara M. R. , and Zaragoza-Maldonado P.-A. D. S. , Traditional Fermented Foods and Beverages From Around the World and Their Health Benefits, Microorganisms. (2022) 10, no. 6, 1–18, 10.3390/microorganisms10061151, 35744669. PMC9227559 35744669

[27] Zugravu C. A. , Bohiltea R. E. , Salmen T. , Pogurschi E. , and Otelea M. R. , Antioxidants in Hops: Bioavailability, Health Effects and Perspectives for New Products, Antioxidants. (2022) 11, no. 2, 10.3390/antiox11020241, 35204124. PMC8868281 35204124

[28] Tamang J. P. , Shin D. H. , Jung S. J. , and Chae S. W. , Functional Properties of Microorganisms in Fermented Foods, Frontiers in Microbiology. (2016) 7, 10.3389/fmicb.2016.00578, 2-s2.0-84966716000, 27199913. PMC4844621 27199913

[29] Alemneh S. T. , Emire S. A. , Jekle M. , and Hitzmann B. , Effect of Refrigerated Storage on Some Physicochemical Characteristics of ateff‐basedfermented Beverage and the Viability of the fermentingLactiplantibacillus *plantarumandLacticaseibacillus* rhamnosusused, Journal of Food Processing and Preservation. (2022) 46, no. 11, e17034, 10.1111/jfpp.17034.

[30] Marshall E. and Mejia D. , Traditional Fermented Food and Beverages for Improved Livelihoods, A Global Perspective, 2012, FAO.

[31] Rolle R. and Satin M. , Basic Requirements for the Transfer of Fermentation Technologies to Developing Countries, International Journal of Food Microbiology. (2002) 75, no. 3, 181–187, 10.1016/S0168-1605(01)00705-X, 2-s2.0-0037172347, 12036141. 12036141

[32] Ashenafi M. , A Review on the Microbiology of Indigenous Fermented Foods and Beverages of Ethiopia, Ethiopian Journal of Biological Sciences. (2006) 5, 189–245.

[33] Abegaz K. , Isolation, Characterization and Identification of Lactic Acid Bacteria Involved in Traditional Fermentation of Borde, an Ethiopian Cereal Beverage, African Journal of Biotechnology. (2007) 6, no. 12, 1469–1478.

[34] Marsh A. J. , Hill C. , Ross R. P. , and Cotter P. D. , Fermented Beverages With Health-Promoting Potential: Past and Future Perspectives, Trends in Food Science & Technology. (2014) 38, no. 2, 113–124, 10.1016/j.tifs.2014.05.002, 2-s2.0-84904403670.

[35] Anteneh T. , Tetemke M. , and Mogessie A. , Antagonism of Lactic Acid Bacteria Against Foodborne Pathogens During Fermentation and Storage of Borde and Shamita, Traditional Ethiopian Fermented Beverages, International Food Research Journal. (2011) 18, no. 3, 1135–1140.

[36] Abegaz K. , Beyene F. , Langsrud T. , and Narvhus J. A. , Indigenous Processing Methods And Raw Materials of borde, an Ethiopian Traditional Fermented Beverage, Journal of Food Technology in Africa. (2002) 7, no. 2, 59–64, 10.4314/jfta.v7i2.19246.

[37] Debebe G. , Determination of Ethanol Level in Beverages, MSc. Thesis, 2006, Addis Ababa University.

[38] World Health Organisation , Global Status Report on Alcohol and Health, 2011, 122, World Health Organisation, https://iris.who.int/bitstream/handle/10665/44499/9789241564151_eng.pdf.

[39] Fahy B. D. , Chapter 2 Published in Changing Modalities of Alcohol Usage, Alcohol in Africa: Mixing Business, Pleasure and Politics, 2002, University of Edinburgh, 22–52.

[40] Andualem B. , Shiferaw M. , and Berhane N. , Isolation and Characterization of *Saccaromyces cervisiae* Yeasts Isolates From “*Tella*” for Beer Production, Annual Research & Review in Biology. (2017) 15, no. 5, 1–12, 10.9734/ARRB/2017/34129, 2-s2.0-85027571032.

[41] Lee M. , Regu M. , and Seleshe S. , Uniqueness of Ethiopian Traditional Alcoholic Beverage of Plant Origin, Tella, Journal of Ethnic Foods. (2015) 2, no. 3, 110–114, 10.1016/j.jef.2015.08.002, 2-s2.0-84943351002.

[42] Binitu W. B. , Indigenous Processing Methods of Cheka: A Traditional Fermented Beverage in Southwestern Ethiopia, Journal of Food Processing & Technology. (2016) 7, no. 1, 10.4172/2157-7110.1000540.

[43] Taye M. , Yilma M. , Rischkowsky B. , Dessie T. , Okeyo M. G. , and Haile A. , Traditional Fermented Drinks: Korefe, Borde, and Booka and Their Safety, Nutritiousness, and Usefulness in Southern Ethiopia, 2023, Research Square, 10.21203/rs.3.rs-3793503/v1.

[44] Sanz-López C. , Amato M. , Torrent D. , Borrego M. , Anza M. , Bibiso M. , Grijalva-Vallejos N. , Vilanova C. , Porcar M. , and Pascual J. , Microbial Ecology of Selected Traditional Ethiopian Fermented Products, Frontiers in Microbiology. (2025) 16, 1570914, 10.3389/fmicb.2025.1570914, 40529576. 40529576 PMC12171217

[45] Gebre T. S. , Emire S. A. , Chelliah R. , Aloo S. O. , and Oh D. H. , Isolation, Functional Activity, and Safety of Probiotics From Ethiopian Traditional Cereal-Based Fermented Beverage, “Borde”, LWT. (2023) 184, 115076, 10.1016/j.lwt.2023.115076.

[46] Hirbo H. and Hola R. , Safety, Nutrition and Functionality of Traditional Fermented Beverages: Korefe, Borde and Booka in Southern Ethiopia, American Journal of Applied and Industrial Chemistry. (2024) 8, no. 1, 23–34, 10.11648/j.ajaic.20250801.13.

[47] Negasi A. , Fassil A. , and Asnake D. , In Vitro Evaluation of Lactic Acid Bacteria Isolated From Traditional Fermented Shamita and Kocho for Their Desirable Characteristics as Probiotics, African Journal of Biotechnology. (2017) 16, no. 12, 594–606, 10.5897/ajb2016.15307.

[48] Bacha K. , Mchari T. , and Ashenafi M. , Microbiology of the Fermentation of shamita, a Traditional Ethiopian Fermented Beverage, SINET: Ethiopian Journal of Science. (1999) 22, no. 1, 113–126, 10.4314/sinet.v22i1.18137.

[49] Kitessa D. A. , Bacha K. , Tola Y. B. , Murimi M. , Gershe S. , and Guta M. , Microbial Quality and Growth Dynamics in Shameta: A Traditional Ethiopian Cereal-Based Fermented Porridge, Fermentation. (2022) 8, no. 3, 10.3390/fermentation8030124.

[50] Fentie E. G. , Jeong M. , Emire S. A. , Demsash H. D. , Kim M. A. , and Shin J. H. , Fermentation Dynamics of Spontaneously Fermented Ethiopian Honey wine, Tej, LWT. (2022) 155, 112927, 10.1016/j.lwt.2021.112927. PMC8933813 35313500

[51] Kuratie G. , The Microbiological and Physicochemical Characteristics of Bubugn, A Traditional Fermented Ethiopian Low Alcoholic Beverage, Biotechnology Journal International. (2021) 11, no. 1, 1–11.

[52] Binitu W. B. , Gemede H. F. , and Woldegiorgis A. Z. , Nutritional and Alcoholic Contents of Cheka: A Traditional Fermented Beverage in Southwestern Ethiopia, Food Science & Nutrition. (2018) 6, no. 8, 2466–2472, 10.1002/fsn3.854, 2-s2.0-85055521212, 30510748. 30510748 PMC6261204

[53] Albene D. , Andeta A. F. , Ali K. , Syraji Y. , Dejene F. , and Kuma S. , Comprehensive Evaluation of Physicochemical Properties and Microbial Dynamics of Cheka: Traditional Fermented Beverage in Southern Ethiopia, International Journal of Microbiology. (2025) 2025, no. 1, 10.1155/ijm/7912854, 41127416, 7912854. 41127416 PMC12539985

[54] Debebe A. , Chandravanshi B. S. , and Redi-Abshiro M. , Assessment of Essential and Non-Essential Metals in Ethiopian Traditional Fermented Alcoholic Beverages, Bulletin of the Chemical Society of Ethiopia. (2017) 31, no. 1, 17–30, 10.4314/bcse.v31i1.2, 2-s2.0-85024502565.

[55] Yohannes T. , Preparation and Physicochemical Analysis of Some Ethiopian Traditional Alcoholic Beverages, African Journal of Food Science. (2013) 7, no. 11, 399–403, 10.5897/ajfs2013.1066.

[56] Yehuala G. A. , Shibeshi N. T. , Kim S. H. , and Park M. K. , Characterization of Autochthonous Lactic Acid Bacteria Isolated From a Traditional Ethiopian Beverage, Tella, Foods. (2024) 13, no. 4, 10.3390/foods13040575, 38397552. PMC10888401 38397552

[57] Amabye T. G. , Evaluation of Phytochemical, Chemical Composition, Antioxidant and Antimicrobial Screening Parameters of *Rhamnus prinoides* (*Gesho*) Available in the Market of Mekelle, Tigray, Ethiopia, Natural Products Chemistry and Research. (2016) 4, no. 1, 10.4172/2329-6836.1000198.

[58] Talema A. and Nega A. , Preparations and Types of Local Traditional Alcoholic Beverage (Tella) in Amhara Region, Amhara, Ethiopia, Journal of Drug and Alcohol Research. (2022) 11, 1–8, 10.4303/jdar/236173.

[59] Kloman H. , A Tale of Two T′allas, 2023, WordPress, http://worldpress.com. https: //ethiopianfood. http://wordpress.com/wp-content/uploads/2024/09/a-tale-of-two-tallas-1.pdf.

[60] Wolde A. and Beriza B. , Fermenter Technology Modification Changes Microbiological and Physicochemical Parameters, Improves Sensory Characteristics in the ermentation of Tella: An Ethiopian Traditional Fermented Alcoholic Beverage, Journal of Food Processing Technology. (2014) 5, no. 4, 2–8, 10.4172/2157-7110.1000316.

[61] Getaye A. , Tesfaye D. , Zerihun A. , and Melese F. , Production, Optimization and Characterization of Ethiopian Traditional Fermented Beverage “Tella” From Barley, Journal of Emerging Technologies and Innovative Research. (2018) 5, no. 4, 795–799.

[62] Tekluu B. , Gebremariam G. , Aregai T. , and Harikrishna R. , Determination of Alcoholic Content and Other Parameters of Local Alcoholic Beverage (Tella) at Different Stages in Gondar, Ethiopia, International Journal of Engineering and Applied Sciences. (2015) 4, 37–40.

[63] Berhanu A. , Microbial Profile of Tella and the Role of Gesho (Rhamnus prinoides) as Bittering and Antimicrobial Agent in Traditional Tella (Beer) Production, International Food Research Journal. (2014) 21, no. 1, 357–365.

[64] Tadesse S. , Chandravanshi B. S. , and Zewge F. , Ethanol, Methanol, Acid Content and Other Quality Parameters of Ethiopian Traditional Fermented, Distilled and Factory Produced Alcoholic Beverages, SINET: Ethiopian Journal of Science. (2017) 40, no. 1, 16–35, 10.4314/sinet.v40i1.

[65] Kassa A. , Investigation of Parabolic Dish Solar Concentrator for Local Areke Distillation. Thesis, 2015, Addis Ababa University.

[66] Tadesse S. and Hailemichael F. , Characterization of Ethiopian Commonly Consumed Traditional Home Distilled Alcoholic Beverages, Global Scientific Research. (2022) 10, no. 1, 2733–2748.

[67] Demissie S. W. , Ramayya V. A. , and Nega D. T. , Design, Fabrication and Testing of Biogas Stove for ‘Areke’ Distillation: The Case of Arsi Negele, Ethiopia, Targeting Reduction of Fuel-Wood Dependence, International Journal of Engineering Research. (2016) 5, 354–365.

[68] World Health Organization (WHO) , Global Status Report on Alcohol, 2004, World Health Organization, Department of Mental Health and Substance Abuse. https: //iris. who.int/bitstream/handle/10665/44499/9789241564151_eng.pdf.

[69] Bahiru B. , Mehari T. , and Ashenafi M. , Chemical and Nutritional Properties of `Tej’, an Indigenous Ethiopian Honey Wine: Variations Within and Between Production Units, Journal of Food Technology in Africa. (2001) 6, no. 3, 10.4314/JFTA.V6I3.19299. 16943014

[70] Berhanu M. , Desalegn A. , Birri D. J. , Mogessie A. , and Fitsum T. , Microbial, Physicochemical and Proximate Analysis of Tej Collected From Amhara Regional State of Ethiopia, Heliyon. (2023) 9, no. 6, e16911, 10.1016/j.heliyon.2023.e16911, 37332921. 37332921 PMC10275989

[71] Bahiru B. , Mehari T. , and Ashenafi M. , Yeast and Lactic Acid Flora of Tej, an Indigenous Ethiopian Honey Wine: Variations Within and Between Production Units, Food Microbiology. (2006) 23, no. 3, 277–282, 10.1016/j.fm.2005.05.007, 2-s2.0-26044466167, 16943014. 16943014

[72] Lyons D. , Tej Consumption and Production in the Commensal Politics and Political Economy of States in Northern Highland Ethiopia, International Journal of Historical Archaeology. (2022) 26, no. 2, 259–290, 10.1007/s10761-021-00597-5.

[73] Gebre T. S. and Emire S. A. , Characterization of “Borde”: A Traditional Ethiopian Cereal-Based Fermented Beverage - Microbial Analysis, Rheological Profiling, and Functional Attributes, Journal of Agriculture and Food Research. (2024) 18, 10.1016/j.jafr.2024.101498, 101498.

[74] Ashenafi M. and Mehari T. , Some Microbiological and Nutritional Properties of Borde and Shamita, Traditional Ethiopian Fermented Beverages, Ethiopian Journal of Health Development. (1995) 9, 1–8.

[75] Nemo R. and Bacha K. , Natural Preservative-Based Shelf-Life Enhancement of Borde: A Traditional Ethiopian Low Alcoholic Beverage, Journal of food Processing and Preservation. (2021) 45, no. 11, e15968, 10.1111/jfpp.15968.

[76] Kitessa D. A. , Bacha K. , Tola Y. B. , and Murimi M. , Preparation and Consumption of Shameta: An Indigenous Cereal-Based Fermented Porridge in Western Ethiopia, East African Journal of Sciences. (2023) 17, 55–70.

[77] Wassie M. and Wassie T. , Isolation, Characterization and Identification of Lactic Acid Bacteria From Ready to Consume Shamita: Ethiopian Traditional Fermented Beverage, International Journal of Life Sciences and Technology. (2016) 9, no. 6, 51–55.

[78] Fentie E. G. , Emire S. A. , Demsash H. D. , Dadi D. W. , and Shin J. H. , Cereal- and Fruit-Based Ethiopian Traditional Fermented Alcoholic Beverages, Foods. (2020) 9, no. 12, 10.3390/foods9121781, 33271792. PMC7761231 33271792

[79] Abawari R. A. , Indigenous Processing Methods and Raw Materials of Keribo: An Ethiopian Traditional Fermented Beverage, Journal of Food Resource Science. (2012) 2, no. 1, 13–20, 10.3923/jfrs.2013.13.20.

[80] Dibaba K. , Tilahun L. , Satheesh N. , and Geremu M. , Acrylamide Occurrence in Keribo: Ethiopian Traditional Fermented Beverage, Food Control. (2018) 86, 77–82, 10.1016/j.foodcont.2017.11.016, 2-s2.0-85042183023.

[81] Hidug D. B. and Mengesha M. G. , Determination of Protein Value and Alcoholic Content in Locally Prepared Different Types of Cheka at Different Stages Using CHNS Elemental Analyzer and Specific Gravity Methods, American Journal of Applied Chemistry. (2019) 7, 168–174, 10.11648/j.ajac.20190706.13.

[82] Tsegaye B. , Girma E. , Agedew E. , Zerihun E. , Hailu T. , Shibru T. , Abebe S. , Kanko T. , and Narayanan V. , Proximal Composition of Indigenous Alcoholic Beverage Cheka in Konso, Southwestern, Ethiopia, Journal of Food Processing Technology. (2020) 11, 1–8, 10.35248/2157-7110.20.11.840.

[83] Gizachew S. , Van Beeck W. , Spacova I. , Dekeukeleire M. , Alemu A. , Mihret W. , Lebeer S. , and Engidawork E. , Characterization of Potential Probiotic Starter Cultures of Lactic Acid Bacteria Isolated From Ethiopian Fermented Cereal Beverages, Naaqe, and Cheka, J Appl Microbiol. (2023) 134, 10.1093/jambio/lxad237. 37858306

[84] Mokoena M. P. , Mutanda T. , and Olaniran A. O. , Perspectives on the Probiotic Potential of Lactic Acid Bacteria From African Traditional Fermented Foods and Beverages, Food & Nutrition Research. (2016) 60, no. 1, 29630, 10.3402/fnr.v60.29630, 2-s2.0-84962252913, 26960543. 26960543 PMC4785221

[85] Debebe A. , Chandravanshi B. S. , and Redi-Abshiro M. , Total Contents of Phenolics, Flavonoids, Tannins and Antioxidant Capacity of Selected Traditional Ethiopian Alcoholic Beverages, Bulletin of the Chemical Society of Ethiopia. (2016) 30, no. 1, 27–37, 10.4314/bcse.v30i1.3, 2-s2.0-84956888528.

[86] Bouakkaz S. , Zerizer H. , Rachedi K. , Accettulli A. , Angela R. A. , and Bevilacqua A. , African Cereal-Based Fermented Foods: Microbiota, Functional Microorganisms, Starter Cultures and Nutritional Properties, Food Bioscience. (2024) 62, 105212, 10.1016/j.fbio.2024.105212.

[87] Achi O. K. and Asamudo N. U. , Cereal-Based Fermented Foods of Africa as Functional Foods, International Journal of Microbiology and Application. (2019) 2, no. 4, 71–83, 10.1007/978-3-319-78030-6_31.

[88] Narayan S. S. , Jalgaonkar S. , Shahani S. , and Kulkarni V. N. , Probiotics: Current Trends in the Treatment of Diarrhoea, Hong Kong Medical Journal. (2021) 16, no. 3. 20519758

[89] Franz C. M. A. P. , Huch M. , Mathara J. M. , Abriouel H. , Benomar N. , Reid G. , Galvez A. , and Wilhelm H. , African Fermented Foods and Probiotics, International Journal of Food Microbiology. (2014) 190, 84–96, 10.1016/j.ijfoodmicro.2014.08.033, 2-s2.0-84907668538. 25203619

[90] Holzapfel W. H. , Appropriate Starter Culture Technologies for Small-Scale Fermentation in Developing Countries, International Journal of Food Microbiology. (2002) 75, no. 3, 197–212, 10.1016/S0168-1605(01)00707-3, 2-s2.0-0037172344, 12036143. 12036143

[91] Mulaw G. , Sisay T. , Muleta D. , and Tesfaye A. , In Vitro Evaluation of Probiotic Properties of Lactic Acid Bacteria Isolated From Some Traditionally Fermented Ethiopian Food Products, International Journal of Microbiology. (2020) 2019, no. 1, 7179514, 10.1155/2019/7179514, 2-s2.0-85072386366, 31534458. PMC6732631 31534458

[92] Aka S. , Camara F. , Nanga Y. Z. , Loukou Y. G. , and Dje K. M. , Evaluation of Organic Acids and Sugars Contents During the Production of " Tchapalo ", A Traditional Sorghum Beer in Côte D′ivoire, Journal of Food Technology. (2008) 6, no. 5, 189–195.

[93] Okereke H. C. , Achi O. K. , Ekwenye U. N. , and Orji F. A. , Antimicrobial Properties of Probiotic Bacteria From Various Sources, African Journal of Biotechnology. (2012) 11, no. 39, 9416–9421, 10.5897/ajb11.3334.

[94] Sawadogo-Lingani H. , Diawara B. , Traoré A. S. , and Jakobsen M. , Technological Properties of *Lactobacillus fermentum* Involved in the Processing of Dolo and Pito, West African Sorghum Beers, for the Selection of Starter Cultures, Journal of Applied Microbiology. (2008) 104, no. 3, 873–882, 10.1111/j.1365-2672.2007.03638.x, 2-s2.0-39149103684, 18031523. 18031523

[95] Tamminen M. , Joutsjoki T. , Sjoblom M. , Joutsen M. , Palva A. E. , and Joutsjoki V. , Screening of Lactic Acid Bacteria From Fermented Vegetables by Carbohydrate Profiling and PCR-ELISA, Letters in Applied Microbiology. (2004) 39, no. 5, 439–444, 10.1111/J.1472-765X.2004.01607.X, 2-s2.0-7044274682, 15482435. 15482435

[96] Lefyedi M. L. , Marais G. J. , Dutton M. F. , and Taylor J. R. N. , The Microbial Contamination, Toxicity and Quality of Turned and Unturned Outdoor Floor Malted Sorghum, Journal of the Institute of Brewing. (2005) 111, no. 2, 190–196, 10.1002/J.2050-0416.2005.TB00665.X, 2-s2.0-27444443934.

[97] Blandino A. , Al-Aseeri M. E. , Pandiella S. S. , Blandino A. , Al-Aseeri M. E. , Pandiella S. S. , Cantero D. , and Webb C. , Cereal-Based Fermented Foods and Beverages, Food Research International. (2003) 366, 527–543, 10.1016/S0963-9969(03)00009-7, 2-s2.0-0038702516.

[98] Aka S. , N’guessan K. F. , Nanga Y. Z. , Loukou Y. G. , Mazabraud A. I. , and Djè K. M. , Characterization of *Lactobacillus* Species Isolated From Mash, Sour Wort and Tchapalo Produced in Côte d′Ivoire, Food. (2010) 4, 49–54.

[99] Maoura N. , Mbaiguinam M. , Nguyen H. V. , Claude G. , and Jacques P. , Identification and Typing of the Yeast Strains Isolated From Bili, A Traditional Sorghum Beer of Chad, African Journal of Biotechnology. (2005) 4, 646–656, 10.5897/ajb2005.000-3117.

[100] Dje M. K. , N′Guessan K. F. , Djeni T. N. D. , and Dadie T. A. , Biochemical Changes During Alcoholic Fermentation in the Production of “Tchapalo”, A Traditional Sorghum Beer, International Journal of Food Engineering. (2008) 4, no. 7, 10.2202/1556-3758.1408, 2-s2.0-55649113115.

[101] Marshall E. , Schreckenberg K. , and Newton A. , Commercialization of Non-Timber Forest Products: Factors Influencing Success. Lessons Learned From Mexico and Bolivia and Policy Implications for Decision-Makers, 2006, UNEP-WCMC.

[102] Singh A. , Singh R. K. , and Sureja A. K. , Cultural Significance and Diversities of Ethnic Foods of Northeast India, Indian Journal of Traditional Knowledge. (2007) 6, 79–94.

[103] Aliguma L. , Magala D. , and Lwasa S. , Uganda: Connecting Small-Scale Producers to Markets: The Case of the Nyabyumba United Farmers Group in Kabale District, 2007, International Institute for Environment and Development.

[104] Kim D. H. , Jeong D. , Song K. Y. , and Seo K. H. , Comparison of Traditional and Backslopping Methods for Kefir Fermentation Based on Physicochemical and Microbiological Characteristics, LWT. (2018) 97, 503–507, 10.1016/j.lwt.2018.07.023, 2-s2.0-85050506647.

[105] Nout M. J. R. , Rombouts F. M. , and Havelaar A. , Effect of Accelerated Natural Lactic Fermentation of Infant Good Ingredients on Some Pathogenic Microorganisms, International Journal of Food Microbiology. (1989) 8, no. 4, 351–361, 10.1016/0168-1605(89)90006-8, 2-s2.0-0024405353, 2701696. 2701696

[106] Moran C. A. , Scholten R. H. J. , Tricarico J. M. , Brooks P. H. , and Verstegen M. W. A. , Fermentation of Wheat: Effects of Backslopping Different Proportions of Pre-Fermented Wheat on the Microbial and Chemical Composition, Archives of Animal Nutrition. (2006) 60, no. 2, 158–169, 10.1080/17450390600562700, 2-s2.0-33645104635, 16649578. 16649578

[107] Ogunremi O. R. , Banwo K. , and Sanni A. I. , Starter-Culture to Improve the Quality of Cereal-Based Fermented Foods: Trends in Selection and Application, Current Opinion in Food Science. (2017) 13, 38–43, 10.1016/j.cofs.2017.02.003, 2-s2.0-85013633277.

[108] Olukotun G. B. , Salami S. A. , Okon I. J. , Ahmadu J. H. , Ajibulu O. O. , and Bello Z. , Assessment of the Effects of Back Sloping on Some Starter Culture Strains and the Organoleptic Qualities of Their Yoghurt Products, Asian Food Science Journal. (2021) 20, no. 9, 29–36, 10.9734/afsj/2021/v20i930340.

[109] Jankhotkaew J. , Casswell S. , Huckle T. , Jankhotkaew J. , Casswell S. , Huckle T. , Chaiyasong S. , and Phonsuk P. , Barriers and Facilitators to the Implementation of Effective Alcohol Control Policies: A Scoping Review, International Journal of Environmental Research and Public Health. (2022) 19, no. 11, 10.3390/ijerph19116742, 35682320. PMC9180061 35682320

[110] Stockwell T. , Giesbrecht N. , Vallance K. , and Wettlaufer A. , Government Options to Reduce the Impact of Alcohol on Human Health: Obstacles to Effective Policy Implementation, Nutrients. (2021) 13, no. 8, 10.3390/nu13082846, 34445006. PMC8399748 34445006

[111] Lachenmeier D. W. , Rehm J. , and Gmel G. , Surrogate Alcohol: What Do We Know and Where Do We Go?, Alcoholism: Clinical and Experimental Research. (2007) 31, no. 10, 1613–1624, 10.1111/j.1530-0277.2007.00474.x, 2-s2.0-34548574946, 17681034. 17681034

[112] Szucs S. , Sarvary A. , McKee M. , and Adany R. , Could the High Level of Cirrhosis in Central and Eastern Europe be Due Partly to the Quality of Alcohol Consumed? An exploratory investigation, Addiction. (2005) 100, no. 4, 536–542, 10.1111/j.1360-0443.2005.01009.x, 2-s2.0-17244371482, 15784068. 15784068

[113] Lee S. B. , Shin J. A. , and Lee K. T. , Determination of Fusel Oil Content in Various Types of Liquor Distributed in Korea, Korean Journal of Food Preservation. (2017) 24, no. 4, 510–516, 10.11002/kjfp.2017.24.4.510, 2-s2.0-85030468517.

[114] Fawell J. , Bailey K. , Chilton J. , Dahi E. , Fewtrell L. , and Magara Y. , Fluoride in Drinking-Water, Background Document for Development of WHO Guidelines for Drinking-Water Quality, 2001, IWA Publishing.

[115] Belete Y. , Chandravanshi B. S. , and Zewge F. , Levels of the Fluoride Ion in Six Traditional Alcoholic Fermented Beverages Commonly Consumed in Ethiopia, Fluoride. (2017) 50, no. 1, 79–96.

[116] Methanol Institute , Adulterated Alcohol Poisoning: Issue Summary, 2013, 26, Methanol Institute.

[117] Osuntogun B. and Aboaba O. , Microbiological and Physico-Chemical Evaluation of Some Non-Alcoholic Beverages, Pakistan Journal of Nutrition. (2004) 3, no. 3, 188–192, 10.3923/pjn.2004.188.192.

[118] Eze V. and Eleke O. , Microbiological and Nutritional Qualities of Burukutu Sold in Mammy Market Abakpa, Enugu State, Nigeria, American Journal of Food and Nutrition. (2011) 1, no. 3, 141–146, 10.5251/ajfn.2011.1.3.141.146.

[119] Amusa N. A. and Odunbaku O. A. , Effect of Processing on Nutritional, Microbiological and Sensory Properties of Kunun-Zaki (a Sorghum Based Non-Alcoholic Beverage) Widely Consumed in Nigeria, Pakistan Journal of Nutrition. (2009) 8, no. 3, 288–292, 10.3923/pjn.2009.288.292.

[120] Fadahunsi I. F. , Ogunbanwo S. T. , and Fawole A. O. , Microbiological and Nutritional Assessment of Burukutu and Pito During Storage, Nature and Science. (2013) 11, 98–103.

[121] Buckner C. A. , Lafrenie R. M. , Denommee J. A. , Caswell J. M. , Want D. A. , Gan G. G. , Leong Y. C. , Bee P. C. , Chin E. , The A. K. H. , Picco S. , Villegas L. , Tonelli F. , Merlo M. , Rigau J. , Diaz D. , Masuelli M. , Korrapati S. , Kurra P. , and Mathijssen R. H. J. , We are Intech Open, the World′s Leading Publisher of Open Access Books Built by Scientists, for Scientists Top 1%, Intech. (2016) 34, no. 8, 57–67.

[122] Fusco A. , Savio V. , Cimini D. , Ambrosio S. , Chiaromonte A. , Schiraldi C. , and Donnarumma G. , In Vitro Evaluation of the Most Active Probiotic Strains Able to Improve the Intestinal Barrier Functions and to Prevent Inflammatory Diseases of the Gastrointestinal System, Biomedicines. (2023) 11, no. 3, 10.3390/biomedicines11030865, 36979844. PMC10046130 36979844

[123] Cuvas-Limon R. B. , Nobre C. , Cruz M. , Rodriguez-Jasso R. M. , Ruiz H. A. , Lored-Trevino A. , Texeira J. A. , and Belmares R. , Spontaneously Fermented Traditional Beverages as a Source of Bioactive Compounds: An Overview, Critical Reviews in Food Science and Nutrition. (2021) 61, no. 18, 2984–3006, 10.1080/10408398.2020.1791050, 32662286. 32662286

[124] Tamang J. P. , Health Benefits of Fermented Foods and Beverages, 2015, CRS Press, 10.1201/b18279, 2-s2.0-85054228106.

[125] Hailu A. T. , The Role of University–Industry Linkages in Promoting Technology Transfer: Implementation of Triple Helix Model Relations, Journal of Innovation and Entrepreneurship. (2024) 13, 10.1186/s13731-024-00370-y.

[126] Jami Y. and Gokdeniz I. , The Role of Universities in the Development of Entrepreneurship, WS Press. (2020) 16, no. 1, 10.24917/20833296.161.7.

[127] Adugna Y. , Mohammed A. , and Tadesse G. , Preparation and Quality Evaluation on Local Beverage (Tella) Prepared With Clay Pot (Insira) and Plastic Jar in North-Eastern Ethiopia, Abyssinia Journal of Science and Technology. (2020) 5, no. 1, 1–8.

[128] Aleffi C. , Tomasi S. , Ferrara C. , Santini C. , Paviotti G. , Baldoni F. , and Cavicchi A. , Universities and Wineries: Supporting Sustainable Development in Disadvantaged Rural Areas, Agriculture. (2020) 10, no. 9, 378–414, 10.3390/agriculture10090378.

[129] Kimaryo V. M. , Massawe G. A. , Olasupo N. A. , and Holzapfel W. H. , The Use of a Starter Culture in the Fermentation of Cassava for the Production of “Kivunde”, A Traditional Tanzanian Food Product, International Journal of Food Microbiology. (2000) 56, no. 2-3, 179–190, 10.1016/S0168-1605(00)00159-8, 2-s2.0-0034212987, 10857544. 10857544

[130] Danse M. , Klerkx L. , Reintjes J. , Rabbinge R. , and Leeuwis C. , Unravelling Inclusive Business Models for Achieving Food and Nutrition Security in BOP Markets, Global Food Security. (2020) 24, 100354, 10.1016/j.gfs.2020.100354.

[131] Wu W. , Zhang A. , van Klinken R. D. , Schrobback P. , and Muller J. M. , Consumer Trust in Food and the Food System: A Critical Review, Foods. (2021) 10, no. 10, 2490–3015, 10.3390/foods10102490, 34681539. 34681539 PMC8536093

[132] Wen L. , Yang L. , Chen C. , Li J. , Fu J. , Liu G. , Kan Q. , Chi-Tang H. , Qingrong H. , Yaqi L. , and Yong C. , Applications of Multi-Omics Techniques to Unravel the Fermentation Process and the Flavor Formation Mechanism in Fermented Foods, Critical Reviews in Food Science and Nutrition. (2024) 64, no. 23, 8367–8383, 10.1080/10408398.2023.2199425, 37068005. 37068005

[133] Galimberti A. , Bruno A. , Agostinetto G. , Casiraghi M. , Guzzetti L. , and Labra M. , Fermented Food Products in the Era of Globalization: Tradition Meets Biotechnology Innovations, Current Opinion in Biotechnology. (2021) 70, 36–41, 10.1016/j.copbio.2020.10.006, 33232845. 33232845

[134] Shi H. , An F. , Lin H. , Li M. , Wu J. , and Wu R. , Advances in Fermented Foods Revealed by Multi-Omics: A New Direction Toward Precisely Clarifying the Roles of Microorganisms, Frontiers in Microbiology. (2022) 13, 1044820, 10.3389/fmicb.2022.1044820, 36590428. 36590428 PMC9794733

