# Correction to: “The Uses, Nutritional Advantages, and Challenges of Traditional Fermented Alcoholic Beverages for Indigenous Communities in Ethiopia”

**DOI:** 10.1155/ijm/9837160

**Published:** 2026-08-17

Fentahun, Mulugeta, The Uses, Nutritional Advantages, and Challenges of Traditional Fermented Alcoholic Beverages for Indigenous Communities in Ethiopia, *International Journal of Microbiology*, 2026, 5679327, 23 pages, 2026. 10.1155/ijm/5679327.

In the published version of the article, the corresponding author’s email address was incorrectly listed as: mulufenta@gmail.com.

The correct corresponding author email address should be:

mulugetafanatahun@dmu.edu.et

We apologize for this error.

